# Implications of ubiquitination and the maintenance of replication fork stability in cancer therapy

**DOI:** 10.1042/BSR20222591

**Published:** 2023-10-03

**Authors:** Donghui Xia, Xuefei Zhu, Ying Wang, Peng Gong, Hong-Shu Su, Xingzhi Xu

**Affiliations:** 1 Shenzhen University General Hospital-Dehua Hospital Joint Research Center on Precision Medicine (sgh-dhhCPM), Dehua Hospital, Dehua, Quanzhou 362500, China; 2 Guangdong Key Laboratory for Genome Stability and Disease Prevention, Carson International Cancer Center, Marshall Laboratory of Biomedical Engineering, Shenzhen University Medical School, Shenzhen University, Shenzhen, Guangdong 518060, China; 3 State Key Laboratory of Agro-biotechnology and MOA Key Laboratory of Soil Microbiology, College of Biological Sciences, China Agricultural University, Beijing, China; 4 Department of General Surgery, Institute of Precision Diagnosis and Treatment of Gastrointestinal Tumors and Carson International Cancer Center, Shenzhen University General Hospital, Shenzhen University Medical School, Shenzhen University, Shenzhen, Guangdong 518060, China

**Keywords:** cancer, replication fork stability, ubiquitination

## Abstract

DNA replication forks are subject to intricate surveillance and strict regulation by sophisticated cellular machinery. Such close regulation is necessary to ensure the accurate duplication of genetic information and to tackle the diverse endogenous and exogenous stresses that impede this process. Stalled replication forks are vulnerable to collapse, which is a major cause of genomic instability and carcinogenesis. Replication stress responses, which are organized via a series of coordinated molecular events, stabilize stalled replication forks and carry out fork reversal and restoration. DNA damage tolerance and repair pathways such as homologous recombination and Fanconi anemia also contribute to replication fork stabilization. The signaling network that mediates the transduction and interplay of these pathways is regulated by a series of post-translational modifications, including ubiquitination, which affects the activity, stability, and interactome of substrates. In particular, the ubiquitination of replication protein A and proliferating cell nuclear antigen at stalled replication forks promotes the recruitment of downstream regulators. In this review, we describe the ubiquitination-mediated signaling cascades that regulate replication fork progression and stabilization. In addition, we discuss the targeting of replication fork stability and ubiquitination system components as a potential therapeutic approach for the treatment of cancer.

## Introduction

DNA replication machinery is controlled by sophisticated regulatory mechanisms to maintain the faithful duplication and transmission of genetic information. However, the accuracy of DNA replication is often challenged by exogenous and endogenous stresses at active replication forks. These include DNA–protein cross-links (DPCs), intra- or inter-strand cross-links, secondary DNA structures, DNA lesions, and imbalanced dNTP pools [1]. Ultimately, replication stress causes chromosome instability, which can in turn lead to carcinogenesis [2]. As a result, the proper regulation and maintenance of DNA replication forks is of critical importance.

When replication stress arises, replication forks may stall in response. Stalling leads to the uncoupling of Cdc45-MCM-GINS (CMG) helicase and DNA polymerase and the generation of single-stranded DNA (ssDNA), which is rapidly bound and protected by the heterotrimeric replication protein A (RPA) complex (RPA1, RPA2, and RPA3). The ensuing recruitment of regulators to RPA-coated ssDNA results in the initiation of replication fork stabilization, replication fork reversal and restart [3], or DNA damage tolerance (DDT) [4]. This also triggers global responses: for example, the activation of cell cycle checkpoints, repression of origin firing, and balancing of the dNTP pool. This is mediated by the ataxia telangiectasia and RAD3-related kinase (ATR)-checkpoint kinase 1 (CHK1) signaling cascade [5].

The replication stress response signaling network is modulated via a series of post-translational modifications, with phosphorylation and ubiquitination being the most important of these. Ubiquitination is a reversible post-translational modification which involves the covalent attachment of ubiquitin (Ub), a compact 76-amino acid protein, on to the lysine residues of various substrates. This modification is catalyzed by three different classes of enzymes: Ub-activating enzymes (E1), Ub-conjugating enzymes (E2), and Ub ligases (E3). During ubiquitination, an E1 enzyme activates the C-terminus of Ub and transfers it to the active cysteine site of an E2 enzyme. Subsequently, an E3 ligase, which specifically recognizes the substrates, acts as an intermediate recruiter of Ub-loaded E2 and catalyzes the ubiquitination of target proteins [6,7]. Polyubiquitination involves the formation of polyubiquitin chains by linking additional Ub molecules together via their lysine residues (K6, K11, K27, K29, K33, K48, or K63) or N-terminal methionine (M1). Meanwhile, attached Ub or Ub chains are removed by the Ub-cleavage activity of deubiquitinases (DUBs) [8].

The location and type of ubiquitination confers different fates to substrates. For example, polyubiquitination via linkage at K48 or K11 marks substrates for degradation via the proteasome system [9]. K63-linked polyubiquitination is non-proteolytic in most scenarios, but has been reported to regulate substrate degradation under restricted circumstances [10]. Furthermore, mono-, multi-mono-, and polyubiquitination at M1, K6, K11, K27, K29, K33, and K63 residues is known to regulate the localization, interactome, and activity of various substrates [9,11]. In particular, the ubiquitination of critical regulators allows for a cascade of molecular interactions and events to counteract replication stress (Tables 1 and 2). For example, RPA ubiquitination promotes the recruitment of the ATR-ATR interacting protein (ATRIP) complex, ensuing activation of the ATR-CHK1 signaling cascade [12], while proliferating cell nuclear antigen (PCNA) ubiquitination is a prerequisite for DDT and the reversal of stalled replication forks [13–15]. Balanced ubiquitination is crucial for the efficient switching of substrate activity and signal transduction during the progression of replication and the resolution of replication stress. Furthermore, if the ubiquitination of regulatory molecules is dysregulated, the stability of replication forks is threatened. When this occurs, chromosome instability may follow, and cancer with it.

**Table 1:** E3 ligases that regulate replication fork stability

E3 ligase	Substrate	Function	REF.
OBI1	ORC	Promotes origin firing and recruitment of DNA replication factors	[16]
SCF-Skp2 DDB1-Cul4	CDT1	Maintains low levels of CDT1 in S and G2 phase	[17]
SPOP	Geminin	Blocks the binding of CDT1 to MCM and prevents the overfiring of replication origins	[18]
SCF^Dia2^	MCM7	Recruits CDC48 to disassemble CMG helicase, mediating termination in budding yeast	[19,20]
CUL2^LRR1^	MCM7	Recruits CDC48 to disassemble CMG helicase, mediating termination in metazoans	[21,22]
SCF^Pof3^	Pol ε/δ MCM-2,-4,-6	Delays replication fork progression via the ubiquitin-proteasome system in Swi1-deficient cells	[23]
PRP19	RPA	Promotes ATR-ATRIP activation	[12]
RFWD3	RPA	Activates ATR-ATRIP and promotes PCNA ubiquitination	[24,25]
HectH9	TopBP1	Reduces TopBP1 recruitment to chromatin and attenuates ATR-dependent signaling	[26]
RAD18	PCNA	Promotes monoubiquitination of PCNA, facilitating binding to TLS polymerase, and helps to bypass problematic lesions	[27]
CRL4^Cdt2^	PCNA	Facilitates the TLS pathway in undamaged cells	[28].
Rad5 HLTF/SHPRH	PCNA	Promotes the TS pathway and recruits the DNA translocase ZRANB3 to stalled replication forks	[13–15]
SCF^FBH1^	RAD51	Prevents reassociation of RAD51 with DNA	[29]
RNF168	H2A	Prevents MRE11-dependent fork degradation	[30]
Bre1	H2B	Facilitates fork stalling and recovery during replication stress	[31,32]
MDM2	PARP1	Increases the activity of RECQ1 and PRIMPOL, promoting restart and repriming of DNA synthesis	[33]
SIAH2	CtIP	Promotes the loading of SIAH2 at stalled replication forks, facilitating efficient restart	[34]
TRAIP	DPCs	Promotes degradation of DPCs via the ubiquitin-proteasome pathway	[35]
TRAIP	CMG	Promotes CDC48/p97-dependent disassembly of CMG at ICLs and NEIL3 pathway	[36,37]
FANCL PHF9	FANCD2/FANCI	Promotes ICL repair via nuclease-dependent DNA incision carried out by XPF (FANCQ)-ERCC1 and SLX4 (FANCP) and diminishes R-loop accumulation	[38,39]

Abbreviations: DPC, DNA–protein cross-link; ICL, DNA interstrand cross-link; TLS, translesion synthesis; TS, template-switching.

**Table 2:** Deubiquitinases that regulate replication fork stability

Deubiquitinase	Substrate	Function	REF.
USP36	PRIMPOL	Enhances PRIMPOL stability at stressed forks	[40]
USP1	PCNA	Removes the monoubiquitin moiety from PCNA to switch off the TLS pathway	[41]
USP1/UAF1	FANCD2	Removes the monoubiquitin moiety from FANCD2 to enable efficient completion of DNA crosslink repair	[42–45]
USP10	PCNA	Cooperates with ISGylated PCNA to suppress the TLS pathway	[46]
USP7	SPRTN	Antagonizes the auto-cleavage and subsequent inactivation of SPRTN	[47]
USP11	SPRTN	Prevents auto-cleavage of monoubiquitinated SPRTN	[48]
VCPIP1/VCIP135	SPRTN	Promotes the relocalization of SPRTN onto chromatin	[49]

Abbreviations: TLS, translesion synthesis; TS, template-switching.

The activation of oncogenes is also known to induce severe replication stress. As a result, this hallmark of cancer may provide various promising targets for the development of therapeutic drugs. In this review, we summarize the ubiquitination modifications that regulate DNA replication and stalled replication fork stabilization, including replication fork reversal and restart, DNA damage tolerance, and the involvement of DNA repair. We also discuss the potential targets yielded by this system for the development of small molecules as anticancer therapies.

## The involvement of ubiquitination in the regulation of replication fork progression

DNA replication comprises several steps, including origin licensing, origin firing, replication fork elongation and termination, and is monitored and regulated by precise, sophisticated cellular machinery. Preparation for DNA replication begins in G1 phase with the recognition of replication origins by the origin recognition complex (ORC), a multi-subunit AAA+ ATPase (ORC1-6 and LRWD1/ORCA in vertebrates) [50]. ORC, together with cell division cycle 6 protein (CDC6), recruits CDC10-dependent transcript 1 (CDT1), which binds to and assembles the minichromosome maintenance 2-7 (MCM2-7) complex. In turn, this complex licenses replication origins. Only a small fraction of origins licensed by pre-replicative complexes (pre-RCs) are fired in S phase. Cyclin-dependent kinase (CDK) and DBF4-dependent kinase (DDK) are responsible for activating the MCM2-7 helicase, which facilitates the formation of CMG helicase and the subsequent unwinding of DNA to form active replication forks [51,52]. The E3 ligase ORC-ubiquitin-ligase-1 (OBI1, also known as C13ORF7/RNF219) catalyzes the multi-monoubiquitination of chromatin-bound ORC3 and ORC5. OBI1 deficiency inhibits the firing of replication origins and recruitment of DNA replication factors such as DNA polymerase α and PCNA; however, pre-RC assembly and origin licensing are unaffected by deficient OBI1-mediated ORC ubiquitination [16].

DNA replication takes place at multiple origins to guarantee efficiency, with replication of genome information occurring only once per cell cycle. Balanced origin firing is essential for the accurate duplication of genomic information: replication overfiring and re-replication increase the likelihood of stalled forks, which can lead to fork collapse if improperly repaired. Thus, origin licensing is carefully regulated and restricted in G1 phase to avoid origin licensing/reactivation after the G1/S transition, and potentially catastrophic outcomes such as amplified replication and chromosome breakage. For example, the expression of CDT1, a pivotal regulator of pre-RC assembly, is subject to strict cell cycle-dependent control. Low levels of CDT1 during S and G2 phases are achieved via the Ub-proteasome system, which is under the regulatory control of the E3 ligases SKP1-cullin1-F-box protein (SCF)-Skp2 and DNA damage-binding protein 1(DDB1)-Cul4 [17]. In S phase, binding of CDT1 to the MCM complex is blocked by geminin protein, preventing the overfiring of replication origins. This process requires the K27-linked polyubiquitination of geminin at K100 and K127 by the E3 ligase SPOP [18]. In prostate cancer, mutant SPOP fails to ubiquitinate geminin, leading to origin overfiring, re-replication, and genomic instability [18,53]. However, geminin can be stabilized by the deubiquitinases DUB3 and ubiquitin carboxyl-terminal hydrolase 7 (USP7), and DUB3 deficiency is known to increase re-replication events [54].

The replisome initiates DNA replication at fired origins by assembling the DNA polymerase α-primase complex via TIMELESS/ TIMELESS-interacting protein (TIPIN)/AND-1 proteins [55]. After the assembly of the CTF18-replication factor complex (RFC) and PCNA, efficient elongation of the leading and lagging strands of replication forks is carried out by DNA polymerase ε and DNA polymerase δ, respectively [56]. Following elongation, the replisome is disassembled during final step of chromosomal DNA replication: termination. This step is accomplished by the degradation of the core component, CMG helicase, which functions to bind and unwind the parental DNA during replication. Once DNA synthesis is terminated through the convergence of opposing replication forks, the CMG helicase is ubiquitinated and disassembled [57,58]. Ubiquitination-mediated destruction of the CMG helicase is catalyzed by the E3 ligase complex SCF^Dia2^ in budding yeast and CUL2^LRR1^ in metazoans [21,19]. The leucine-rich repeat domains of Dia2 and LRR1 bind to the zinc-finger domains of MCM3 and MCM5 within the CMG complex, but this interaction is occluded by the excluded DNA strand at replication forks before termination [59]. In budding yeast, ubiquitination of CMG helicase on MCM7 K29 residues occurs at the end of DNA replication. This ubiquitination signal is recognized by the UFD1-NPL4 complex, which recruits the segregase cell division control protein 48 (CDC48, also known as p97) to drive the disassembly of CMG helicase [20]. CUL2^LRR1^-mediated ubiquitination of MCM7 also drives the disassembly of CMG helicase by UFD1-NPL4-CDC48 in S phase. Moreover, a backup pathway involving CDC48 and its cofactor UBXN-3 is activated during prophase, where it removes the accumulated CMG helicase on chromatin under conditions of CUL2 ^LRR1^ deficiency [22].

In addition to the replisome, the replication fork protection complex (FPC), exemplified by the Swi1-Swi3 complex in fission yeast, moves with the replication forks and acts to stabilize them [60]. Importantly, Swi1 deficiency triggers the destruction of DNA polymerases (Polε and Polδ) and DNA helicases (MCM-2,-4,-6) via the ubiquitin-proteasome system, which is regulated by the E3 ligase SCF^Pof3^. This degradation delays replication fork progression, avoiding aberrant DNA replication and mitotic catastrophe [23]. In general, ubiquitination and deubiquitination targeting the core components of the replisome or related factors, which regulate the assembly or disassembly of the replication machinery in cell cycle-dependent manner, contribute to the precise progression of replication forks.

## Ubiquitination coordinates a concerted response at stalled replication forks

**Figure 1:**
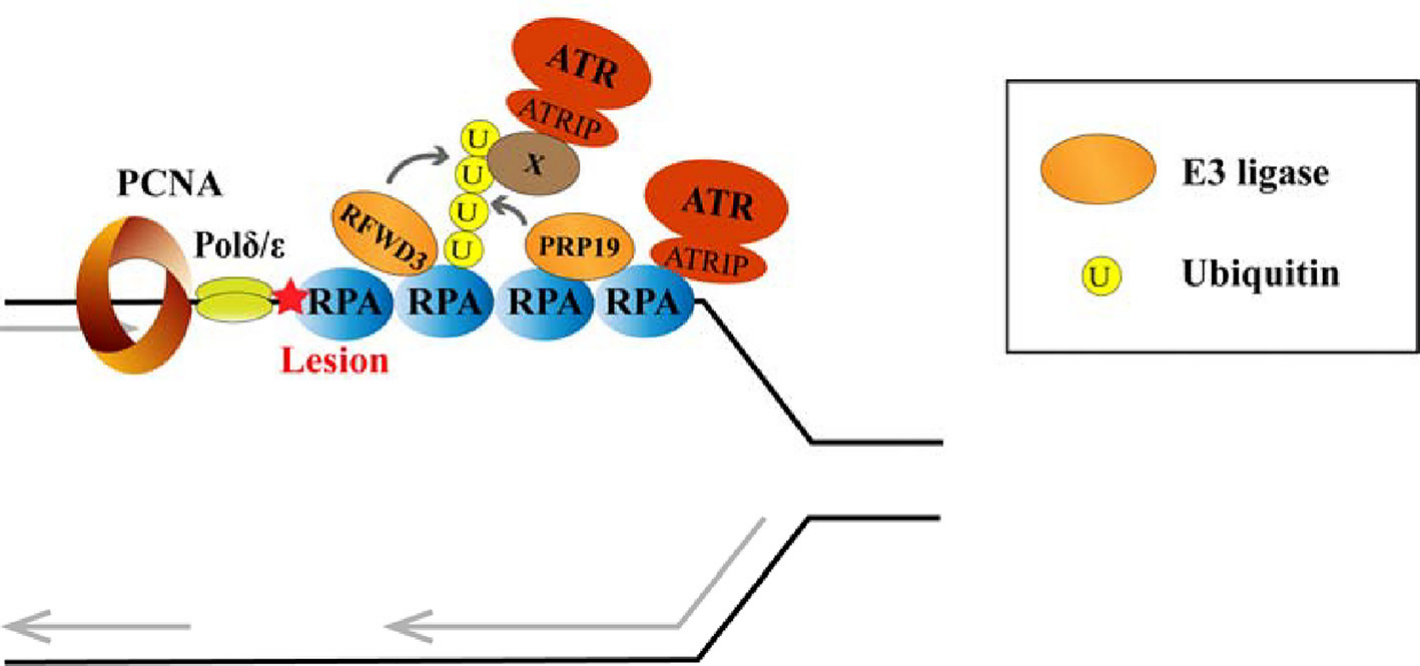
Ubiquitination at the RPA-coated ssDNA platform under replication stress ATR activation promotes PRP19 recruitment to RPA-coated ssDNA, PRP19 and RFWD3 catalyze RPA poly-ubiquitination at the stalled replication fork. ATR-ATRIP is recruited to stalled replication forks by directly binding to RPA. Recruitment of ATR-ATRIP is also promoted by RPA ubiquitination, and that might be mediated by an uncharacterized protein (protein X) as the lack of ubiquitin binding domain on ATR-ATRIP.

Replicating DNA is predisposed to collapse if it is insufficiently stabilized or unable to efficiently counteract threats that impede its progression. Stalling DNA polymerases are uncoupled from CMG helicase, leading to the generation of ssDNA that is rapidly bound and protected by the RPA complex. RPA-coated ssDNA serves a platform for the loading of downstream regulators capable of responding to replication stress. Among these factors, ATR kinase is a key regulator that stabilizes stalled replication forks and orchestrates a global response to trigger cell cycle checkpoints, repress origin firing, and sustain a balanced dNTP pool [61]. ATR knockout leads to chromosome breakage and cell death, while ATR haploinsufficiency and mutation cause tumorigenesis [62,63]. ATR, together with ATR-interacting protein (ATRIP), is recruited to stalled replication forks by RPA [64]. Although ATR-ATRIP can directly bind to RPA-ssDNA, it is proposed that optimal loading of the ATR-ATRIP complex on to stalled replication forks also requires K63-linked polyubiquitination of RPA, which is mediated by the E3 ligase pre-mRNA-processing factor 19 (PRP19) (Figure 1) [12]. However, whether ATR-ATRIP could directly recognize this K63-linked polyubiquitination on RPA still need further elucidation. Moreover, the lack of ubiquitin binding domains on the reported ATR-ATRIP cryo-EM structure raises the possibility of the existence of a potential protein X that bridges the connection between ATR-ATRIP and the ubiquitin chains [12]. The replication stress/DNA damage-induced interaction between PRP19 and RPA is dependent on PI3K kinase (ATR, ATM, and DNA-PK)-mediated phosphorylation of RPA, forming a feed-forward loop that accelerates ATR activation [12,65]. In addition, the E3 ligase RFWD3 interacts constitutively with RPA, promoting the optimized ubiquitination of RPA and the activation of ATR [24]. Notably, recruitment of ATR-ATRIP is essential for the activation of ATR kinase, but is not in itself sufficient: ATR kinase activation also requires the allosteric activators topoisomerase II binding protein 1 (TopBP1) and Ewing tumor-associated antigen 1 (ETAA1). TopBP1 and ETAA1 are recruited to stalled replication forks via RFC-RAD17/RAD9-RAD1-HUS1 complexes and RPA, respectively [66,67]. Following its recruitment to chromatin by MIZ1, TopBP1 is protected from proteasomal degradation mediated by the ubiquitin ligase HectH9 (Mule, ARF-BP1, and HUWE1); however, the oncoprotein MYC disrupts the association between TopBP1 and MIZ1. This results in reduced TopBP1 levels and attenuated ATR-dependent signaling [26].

**Figure 2:**
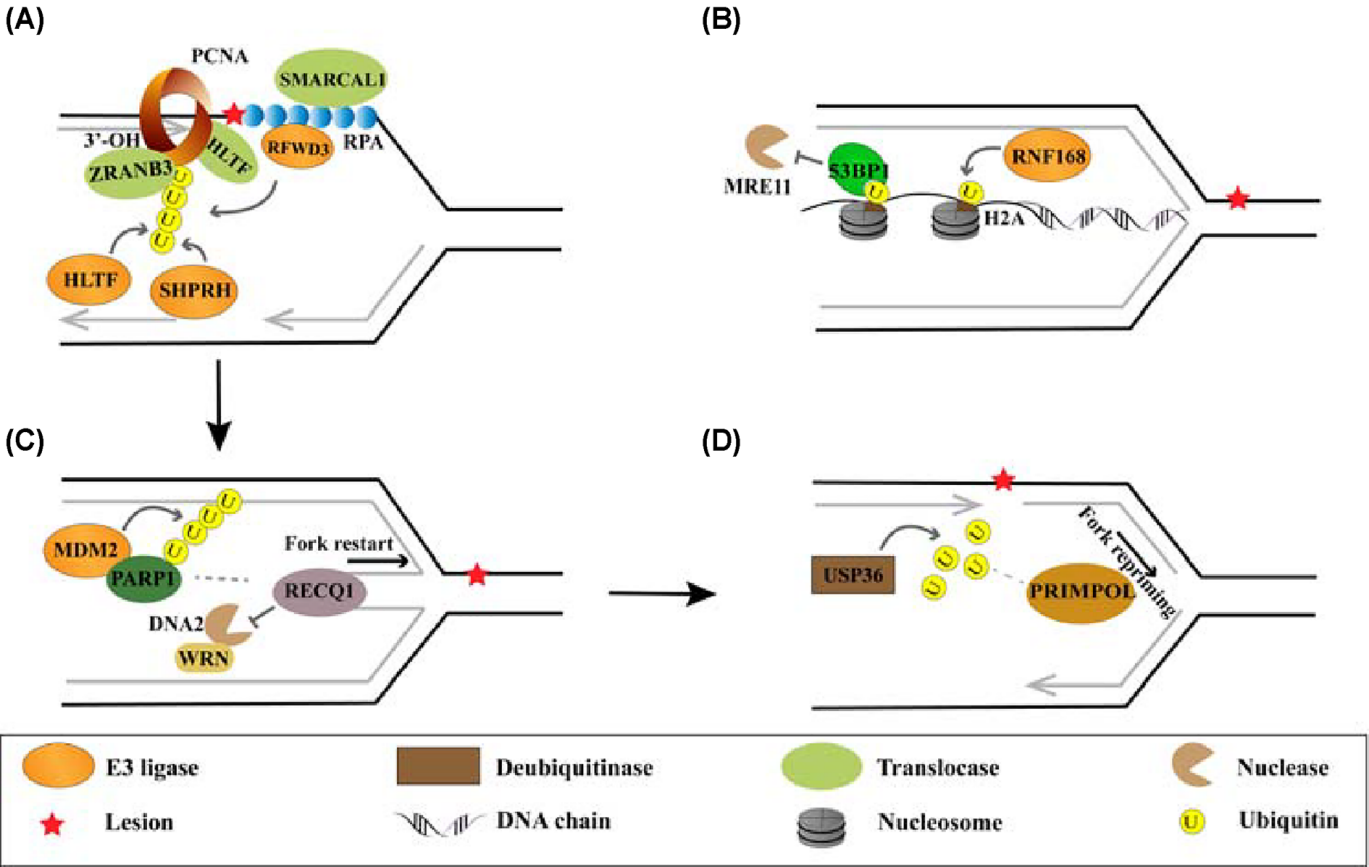
Regulation of fork reversal and restart by ubiquitination (**A**) Fork reversal is initiated by DNA translocases such as SMARCAL1, ZRANB3, and HLTF. SMARCAL1 binds to RPA-coated ssDNA, while ZRANB3 is recruited at stalling forks via polyubiquitinated PCNA mediated by RFWD3, HLTF, or SHPRH, and HLTF has an affinity for available 3′-OH groups located near the fork junction. These three translocases may act on different types of stalled forks or perform distinct functions at the same fork. (**B**) The regressed arm of a reversed fork requires protection against nucleolytic attacks. RNF168-mediated monoubiquitination of H2A on K13/15 at stalled forks is recognized by 53BP1, protecting the regressed arms from degradation by MRE11: this modification also regulates nucleosome deposition, facilitating efficient fork restart. (**C**) Replication fork restart or repriming requires specific factors such as RECQ1 and PRIMPOL. RECQ1 activity is increased following the ubiquitination and inactivation of PARP1 by the oncogenic E3 ligase MDM2, which accelerates DNA replication progression. (**D**) Deubiquitination of PRIMPOL by USP36 enhances its stability at stressed forks, contributing to replication repriming.

Under normal conditions, RFWD3, which promotes the ubiquitination of RPA, is reported to localize at replication forks and is essential for efficient DNA replication, and it is suggested that RFWD3 loads onto replication forks via interaction with PCNA [68]. Under conditions of fork collapse, the RPA-mediated localization of RFWD3 to ssDNA is critical for gap-filling repairs [25, 69, 70]. The interaction between RFWD3 and RPA can be disrupted by the mutation of RFWD3 in the WD40 repeat region (I639K) or by single point mutations in the RPA32 subunit of RPA (F248, E252, or H254), which inhibits the recruitment of RFWD3 to DNA damage sites [70]. RFWD3 also promotes the ubiquitination of PCNA and the ensuing recruitment of downstream effectors (Figure 2A) [25]. Similar to RPA-coated ssDNA, polyubiquitinated PCNA serves as a platform for concerted molecular events to stabilize stalled replication forks. PCNA is monoubiquitinated at lysine 164 by the E3 ligase RAD18, which is recruited to stalled replication forks by RPA [27]. Further K63-linked polyubiquitination of PCNA at K164, which is catalyzed by Rad5 in yeast or by its homologs helicase-like transcription factor (HLTF) and SNF2 histone-linker PHD-finger RING-finger helicase (SHPRH) in mammalian cells [71,72], leads to the recruitment of the DNA translocase zinc finger RANBP2-type containing 3 (ZRANB3) to stalled replication forks [13,14]. HLTF has a strong preference for available 3′-OH groups near fork junctions, and in addition to its E3 ligase activity, exhibits translocase activity that contributes to replication fork remodeling [73]. Meanwhile, another translocase, SWI/SNF-related, matrix-associated, actin-dependent regulator of chromatin, subfamily A like 1 (SMARCAL1), is loaded via interaction with RPA [74]. The DNA translocases SMARCAL1, ZRANB3, and HLTF initiate the reversal of stalled replication forks, forming a protective four-way junction (also termed the ‘chicken foot’) to overcome replication stress (Figure 2A). An *in vitro* study showed that SMARCAL1, ZRANB3, and HLTF recognize different fork structures, indicating a need for different remodelers to cope with the diverse fork structures generated by challenges to replication.

The regressed arms at fork reversal sites resemble free DNA double-strand break (DSB) ends and, as such, are potentially vulnerable to attack by nucleases such as DNA2, exonuclease 1 (EXO1), C-terminal binding protein interacting protein (CtIP), and meiotic recombination 11 homolog 1 (MRE11). The recruitment of specific DSB repair regulators to reversed forks is, therefore, necessary to protect the DNA from nucleolytic threats [30]. For example, the formation of BRCA2-dependent RAD51 filaments at stalled replication forks protects the nascent DNA from MRE11-, MUS81-, CtIP-, and EXO1-mediated degradation; this protective effect partially underlies the chemosensitivity of BRCA2-deficient tumors [75,76]. Ubiquitination of RAD51 by the E3 ligase SCF^FBH1^ complex at K58 and K64 also serves to prevent any unregulated activity [29]. The E3 ligase RNF168, a key regulator in DSB repair, is also localized at replication forks, and its deficiency leads to elevated fork reversal. RNF168-mediated monoubiquitination of H2A on K13/15 at stalled forks leads to recognition by the DSB repair protein 53BP1, which promotes non-homologous end joining-mediated repair and counteracts the homologous recombination (HR)-mediated repair of DSB. This protects the regressed arms from degradation by MRE11, and regulates nucleosome deposition for efficient fork restart (Figure 2B) [30]. In addition, PCNA ubiquitination at K164 results in the recruitment of the translocase ZRANB3, which promotes fork reversal and protects stalled forks from nucleolysis (Figure 2A). Mutations in K164R cause enhanced retention of PCNA on chromatin, leading to aberrant nucleosome packaging and susceptibility to degradation by DNA2 at stalled replication forks [77].

The prompt restart of reversed forks following the resolution of replication threats is necessary for the effective completion of DNA synthesis. The restarting of reversed forks is promoted by the human helicase RECQ1 in a DNA2- and WRN-dependent manner, with limited degradation of nascent DNA [78]. As a key contributor to the DNA damage response, PARP1 also participates in the regulation of stalled replication fork reversal and restart. It is proposed that PARP1 binds to stalled replication forks and recruits the translocases SMARCAL1, ZRANB3, and HLTF to initiate fork reversal [79]. The PARylation activity of PARP1 further prevents the RECQ1-dependent premature restart of reversed forks [80]. PARP1 also recruits MRE11 to stalled replication forks, promoting end resection and efficient fork restart [81]. The oncoprotein mouse double minute 2 homolog (MDM2), a well-known E3 ligase of tumor suppressor P53, is known to ubiquitinate PARP1, targeting it for proteasomal degradation. Correspondingly, elevated levels of MDM2 destabilize PARP1, reduce stalled replication fork reversal, and induce nascent DNA elongation as mediated by the RECQ1 and primase/polymerase PRIMPOL. These effects ultimately lead to replication fork instability and micronuclei accumulation (Figure 2C) [33]. As a mechanism of alleviating replication stress, the deubiquitination of PRIMPOL at K29-linked polyubiquitination sites is carried out by USP36, leading to PRIMPOL stabilization. Replication stress enhances the interaction between USP36 and PRIMPOL, but this is dependent on the deubiquitination of USP36 at lysine 329 and lysine 338 (Figure 2D) [40]. Moreover, the E3 ligase SIAH2 interacts with and ubiquitinates CtIP at K62, K78, K115, K132, and K133, which promotes the loading of CtIP at stalled replication forks and facilitates efficient fork restart. [34].

The particular structure of stalled replication forks determines the finely tuned responses orchestrated by multiple molecular events involved in different repair pathways. The regressed arms should be protected from nucleases as its resemblance with free DSB ends, moreover, pathway choice with DNA damage tolerance is also noteworthy as it also depends on PCNA ubiquitination at K164.

## DNA damage tolerance (DDT) pathway

**Figure 3:**
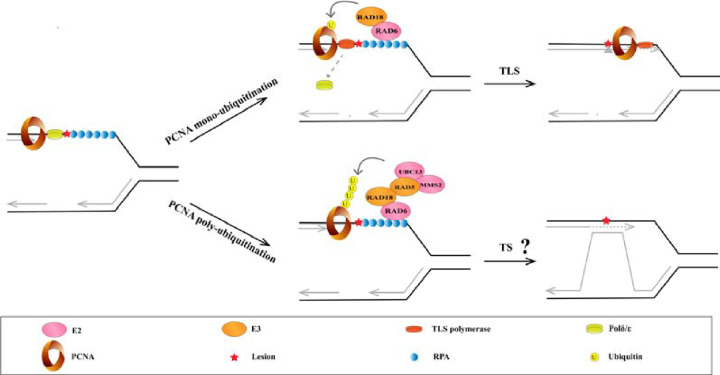
PCNA ubiquitination in the translesion synthesis and template switching pathways PCNA monoubiquitination promotes polymerase switching to activate the translesion synthesis pathway (TLS) and enable the bypassing of problematic lesions. Under replication stress, RPA-coated ssDNA recruits a complex of RAD18 and RAD6, leading to PCNA monoubiquitination at K164. This modification disrupts the affinity of PCNA for B-family DNA polymerases (Polδ and Polε) and increases its affinity for specialized Y-family polymerases (including Polη, Polι, Polκ, and Rev1); these events lead to error-prone TLS. In template switching (TS), following the initial RAD6/RAD18-dependent monoubiquitination of PCNA at K164, UBC13-MMS2, and RAD5 are required to generate K63-linked polyubiquitin chains. However, the exact details of TS following PCNA polyubiquitination remain unknown.

To support the progression of DNA replication forks, RPA-coated ssDNA localized at stalled forks triggers the DDT pathway. This pathway includes error-prone translesion synthesis (TLS) and error-free template switching (TS), which are driven by PCNA ubiquitination [82,83]. Under replication stress, RPA-coated ssDNA recruits E3 ligase RAD18 and E2 enzyme RAD6, leading to PCNA monoubiquitination at K164 (Figure 3) [27,84–87]. This modification alters PCNA conformation, disrupting its affinity for B-family DNA polymerases (Polδ and Polε) and increasing its affinity for the specialized Y-family polymerases (including Polη, Polι, Polκ, and Rev1), which carry out error-prone TLS [83,88]. Although the Y-family polymerases are characterized by both low processivity and low fidelity, Polη can accurately replicate thymidine-thymidine dimers and bypass UV-induced cyclobutane pyrimidine dimers [89]. Polη deficiency causes the genetic disorder xeroderma pigmentosum variant, which causes predisposition to sunlight-induced skin cancer [90]. In addition to the putative RAD18/RAD6-catalyzed monoubiquitination of PCNA described above, PCNA is monoubiquitinated at K164 by the E3 ligase complex CRL4^Cdt2^ in unperturbed cells [28]. However, to ensure replication accuracy, monoubiquitination by CRL4^Cdt2^ is constitutively antagonized by ubiquitin-specific protease 1 (USP1)-mediated deubiquitination [28]. Conversely, UV-induced auto-cleavage of USP1 enhances the accumulation of monoubiquitinated PCNA, promoting TLS [41]. At the termination of TLS, monoubiquitinated PCNA binds to EFP, an E3 ligase for the ubiquitin-like protein interferon-stimulated gene 15 (ISG15). EFP-catalyzed ISGylation of PCNA leads to recognition by the deubiquitinase USP10, which removes the monoubiquitin moiety from PCNA and allows the release of the Y-family polymerases. Subsequently, deISGylation by USP43 enables PCNA to rebind to replicative DNA polymerases in order to resume normal DNA replication [46]. In addition, the E3 ligases HERC2 and RNF8 promote TLS across abasic sites at (or close to) stalled replication forks in a monoubiquitinated PCNA-independent manner [91]. However, the exact targets of HERC2 and RNF8 remain to be determined.

As well as mediating the transition from replicative to TLS polymerases, ubiquitinated PCNA also recruits other regulators, including the AAA+ class ATPase Werner helicase interacting protein 1 (WRNIP1), which has multiple functions. WRNIP1 (Mgs1 in yeast) contains a ubiquitin-binding zinc finger (UBZ) domain, which directs the binding of WRNIP1 to ubiquitinated PCNA and disrupts the association between PCNA and polymerase δ at stalled replication forks [92,93]. Simultaneously, WRNIP1 promotes stabilization of RAD51, preventing uncontrolled nucleolytic attack by MRE11 [94,95]. During replication stress, WRNIP1 also bridges ATM signaling by interacting with ATM interactor protein (ATMIN), thereby preventing aberrant mitotic segregation [96].

In contrast to TLS, TS uses the undamaged nascent sister chromatid as a template to carry out limited DNA replication [97]. This process occurs after fork reversal, enabling the bypassing of DNA lesions, and is dependent on PCNA polyubiquitination. K63-linked polyubiquitination of PCNA mediated by UBC13-MMS2/RAD5 (an E2, E2 variant, and E3 ubiquitin ligase, respectively) in yeast (Figure 3) follows its initial RAD6/RAD18-dependent monoubiquitination at K164 [98,99]. In mammalian cells, two human RAD5 homologs, HLTF and SHPRH, are responsible for the K63-linked polyubiquitination of PCNA at K164 [71,72]. While helicase-coupled repriming occurs downstream of the DNA lesions for continued replication fork progression, the DNA lesions and gaps left behind replication forks due to repriming are reported to be resolved accurately by template switch [100]. In *Saccharomyces cerevisiae*, Polα/Primase/Ctf4 deficiency disturbs the scheduled repriming, leading to longer ssDNA, increased fork reversal, decreased error-free DTT, and increased error-prone DTT. Although long stretch of ssDNA seems to repress error-free DTT, it is required for the loading of recombinase RAD51 and PCNA monoubiquitination E3 ligase RAD18. More precise mechanisms keep the delicate balance that guide the response at stalled replication forks to fork repriming, fork reversal, and error-free or error-prone DTT worth further elucidation. The entire procedure of template switching is not restricted in S phase. TS intermediates pseudo-double Holliday-Junctions may persist till G2/M phase and is processed by Mus81-Mms4 and Slx4 nucleases in cell cycle-dependent manner [101–104].

TS-dependent K63-linked polyubiquitination is elongated at the basis of PCNA monoubiquitination at K164 residue which mediates TLS. Inhibition of this type of PCNA polyubiquitination is known to skew DNA damage tolerance toward TLS and to increase UV-induced mutagenesis [105]. Ready access to the recombination template of TS due to sister chromatid cohesion established during DNA replication and the recruitment of recombinase RAD51 provide the preference of TS over TLS during S phase [101,106]. Besides, chromatin status and chromosome architecture are both proposed to regulate the DDT pathway choice [97,107–109].

PCNA is also modified by the attachment of the small ubiquitin-like modifier (SUMO) at K164 site. SUMOylated PCNA is recognized by the helicase Srs2, which represses HR by disrupting RAD51 filaments in yeast [110,111]. RAD51-mediated HR represents an alternative mechanism for bypassing DNA lesions. PCNA SUMOylation represses unscheduled recombination and helps maintain the balance of DDT and HR at stalled replication forks [112]. In a DDT-deficient scenario, DNA DSBs generated at collapsed replication forks are repaired via HR to initiate DNA replication. Monoubiquitination of histone H2B (H2Bub) at K123, catalyzed by Bre1, is critical for both the DDT and HR pathways during replication stress. In the absence of H2Bub, cells are more susceptible to hydroxyurea-induced replication stress, exhibiting increased RPA foci and decreasing Rad51 recruitment in S phase [31,32]. Another anti-recombination helicase, FBH1, which also functions as an F-box E3 ligase that targets RAD51 for degradation, acts as a master regulator of HR by limiting RAD51-mediated recombination at blocked replication forks [113]. FBH1 is recruited by PCNA via PCNA-interacting peptide (PIP), leading to repressed HR at replication forks. Subsequently, the PIP box is recognized by the E3 ligase complex CRL4^Cdt2^, triggering the ubiquitin-proteasome degradation of FBH1 and ensuing recruitment of TLS polymerases [114]. As a highly conserved site, K164 residue of PCNA is subjected to three post-translational modifications, namely SUMOylation, monoubiquitination, and polyubiquitination. The recombinase RAD51 also plays pivotal role in both DDT and HR pathways at stalled replication forks. The multifunction of these key players reflects the delicate balances of pathway choice according to specific scenarios.

## Engagement of DNA repair pathways

**Figure 4:**
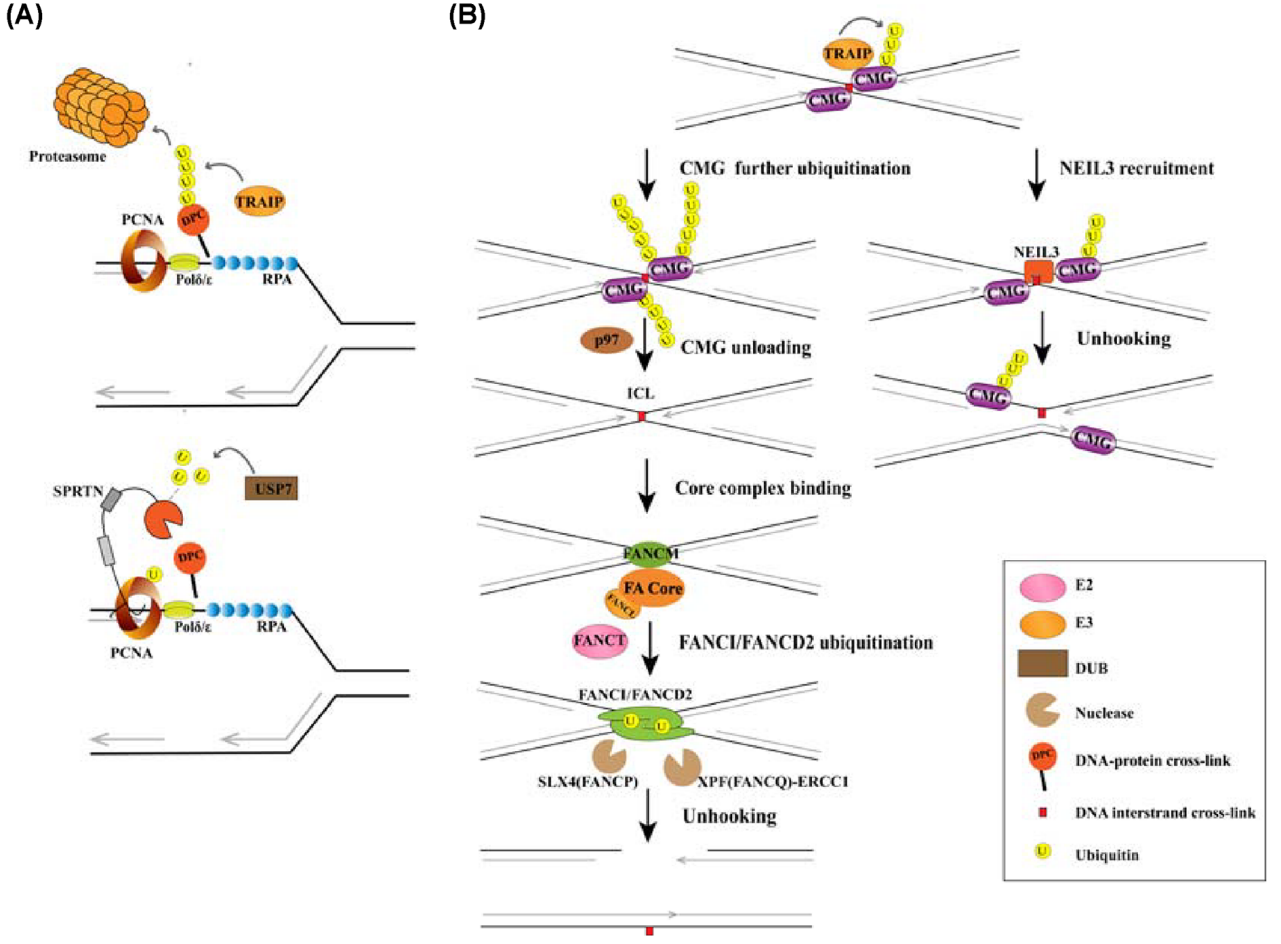
The role of ubiquitination in the removal of DNA-protein cross-links and interstrand cross-links (**A**) The removal of DNA–protein cross-links (DPCs) relies mainly on TRAIP/proteasomes (top), which degrade polyubiquitinated DPCs, or SPRTN (bottom), which is recruited by ubiquitinated PCNA and deubiquinated by USP7. (**B**) Interstrand cross-link (ICL) removal is mainly mediated by the FA pathway. When two replication forks converge on an ICL, TRAIP-generated Ub chains recruit the glycosylase NEIL3 to directly cleave cross-links, while longer Ub chains promote CDC48/p97-dependent CMG disassembly. Next, a heterotetrameric FANCM complex recognizes the stressed replication forks and recruits the FA core complex. Within the core complex, FANCL (E3 ubiquitin ligase) and FANCT (E2) monoubiquitinate FANCD2 at K561 and FANCI at K523. This step promotes the nuclease-dependent DNA incision by XPF (FANCQ)-ERCC1 and SLX4 (FANCP), leading to the unhooking of ICLs and generation of DSB intermediates.

In addition to becoming uncoupled from DNA polymerase at stalled replication forks, CMG helicase can also be blocked when encountering specific DNA lesions. Such lesions include DPCs and DNA interstrand cross-links (ICLs). The proteasome-dependent removal of DPCs relies partially on E3 ligase TRAIP-mediated DPC polyubiquitination during DNA replication (Figure 4A) [35]. DPCs can also be degraded by the metalloprotease SPARTAN (SPRTN), independent from the ubiquitin-proteasome system (Figure 4A). Germline SPRTN mutations cause Ruijs-Aalfs syndrome, which is characterized by premature aging and hepatocellular carcinoma [115,116]. Monoubiquitination by an unknown E3 ligase triggers the autocleavage and proteasomal degradation of SPRTN; however, under DPC-induced replication stress conditions, the DUBs USP7 and USP11 are reported to deubiquitinate SPRTN following its localization to chromatin [47,48]. The DUB VCPIP1/VCIP135 also deubiquitinates SPRTN after being phosphorylated and activated by ATM/ATR. The deubiquitination of SPRTN promotes its acetylation and ability to localize to chromatin [49]. As SPRTN acts on loosely folded DPCs, ubiquitin-modified DPCs must first be unfolded by p97 in association with the ubiquitin adapter complex Ufd1-Npl4 to facilitate SPRTN-mediated proteolysis [117]. Moreover, SPRTN also recognizes ubiquitinated PCNA, which promotes the recruitment of RAD18 and removal of USP1 from chromatin. This further accelerates PCNA monoubiquitination [118]. However, further investigation into the mechanisms underlying the resolution of DPCs is still required. For example, although the TRAIP-proteasome system and SPRTN-dependent pathway are known to be involved in DPC removal, the functions of other DPC proteases in response to different types of DPCs should also be characterized. In addition, while ubiquitination and deubiquitination are known to function as molecular switches of SPRTN activity, the corresponding E3 ligase is yet to be identified.

ICLs halt replication fork progression and transcription by covalently connecting the two strands of DNA [119]. Defective ICL repair causes Fanconi anemia (FA), an autosomal recessive disorder characterized by developmental abnormalities, bone marrow failure, and predisposition to cancers such as leukemias and carcinomas [120–122]. The DNA replication-dependent FA pathway, which involves ubiquitination of the core component FANCD2/FANCI, is well known for its critical role in ICL repair (Figure 4B). Unloading of the CMG helicase from chromatin is a prominent event in the initiation of the FA pathway, in a similar manner to replication termination. Replication termination involves the polyubiquitination of MCM7 and CDC48/p97-dependent disassembly of CMG, while polyubiquitination of CMG at ICLs requires TRAIP but not CUL2^LRR11^ [36,37]. Short TRAIP-generated Ub chains recruit the glycosylase NEIL3 to directly cleave cross-links, while longer Ub chains promote p97-dependent CMG unloading [36]. Subsequently, the anchoring complex, which consists of the ATPase FANCM, FAAP24, MHF1, and MHF2, recognizes the stalled replication fork and mediates recruitment of the FA core complex [119,123,124]. The E3 ubiquitin ligase FANCL (PHF9) then cooperates with FANCT (UBE2T), an E2 enzyme, to monoubiquitinate FANCD2 and FANCI on K561 and K523, respectively [125–127]. Intriguingly, FANCM complex-deficient cells exhibit only partially reduced levels of mono-ubiquitinated FANCD2/FANCI [124], suggesting the existence of an alternative FA core complex recruitment pathway. Consistent with this notion, UHRF1 has been found to bind to ICLs and promote the recruitment of FANCD2 [128]. Ubiquitination of the FANCI/FANCD2 complex promotes nuclease-dependent DNA incision by XPF (FANCQ)-ERCC1 and SLX4 (FANCP) [38, 129, 130], leading to the unhooking of ICLs. The resulting intermediate DSBs are then repaired via HR, classical nonhomologous end joining, alternative nonhomologous end joining and single-strand annealing [120]. To bypass DNA lesions, the unhooking step also includes the recruitment of the TLS polymerases Rev1 and Polι [131]; however, the adducts remaining in the parental strand cannot be removed completely [132]. FANCD2 is also recruited to R-loops, which are DNA:RNA hybrid structures with displaced ssDNA, via binding the displaced ssDNA strand and ssRNA tail. Monoubiquitinated FANCD2/FANCI is also known to promote the resolution of R-loops, but the precise mechanism remains elusive [39].

The TLS and FA pathways are closely interconnected. For example, RAD18/RAD6 is required for efficient FANCD2/FANCI monoubiquitination and chromatin localization [133,134]. Meanwhile, FAAP20, an FA complex subunit responsible for core complex stability and FANCD2 monoubiquitination, binds to monoubiquitinated Rev1 via its Ub-binding zinc finger 4 domain and stabilizes Rev1 nuclear foci [135,136]. In addition, monoubiquitinated FANCD2/FANCI proteins are deubiquitinated by the USP1/UAF1 complex after DNA removal, completing the ICL repair procedure [42–45].

## Targeting ubiquitination and replication fork stability in cancer therapy

Drug resistance is one of the major challenges faced when developing treatment strategies for various types of cancer. The ubiquitin–proteasome system (UPS) regulates nearly all cellular activities as it controls protein turnover, and aberrant expression levels of oncoproteins or tumor suppressors increase the likelihood of carcinogenesis. As a result, the UPS represents an emerging target for the development of novel anticancer drugs and chemosensitizers, with the aim of overcoming drug resistance. This approach has already borne fruit: bortezomib, the first therapeutic proteasome inhibitor to be approved by the Food and Drug Administration (FDA) for the treatment of multiple myeloma (MM) [137] and mantle cell lymphoma (MCL) [138], exhibits favorable selectivity towards tumor cells over normal cells. Bortezomib functions by inhibiting chymotrypsin-like activity, primarily through its interactions with the proteasome β5 core particle [139]. Bortezomib retreatment in patients with myeloma has no cumulative toxicity, and a combination of bortezomib with a rituximab, cyclophosphamide, doxorubicin, and prednisone treatment regimen (VcR-CAP) has shown improved outcomes compared with the R-CHOP regimen [138]. This latter regimen involves a combination of bendamustine plus rituximab with rituximab plus cyclophosphamide, doxorubicin, vincristine, and prednisone. However, bortezomib has no obvious effect in on solid tumors such as prostate cancer, breast cancer, and non-small cell lung cancer [140]. The potency of bortezomib and apparent lack of treatment resistance has driven the development of next generation proteasome inhibitors, such as carfilzomib and ixazomib, for the treatment of blood cancers. More inhibitors are in the preclinical investigation phase, or are currently undergoing clinical trials (Table 3).

**Table 3:** Small molecules designed to target proteasomes for cancer therapy

Target	Inhibitor	Cancer types	Phase	REF.
Proteasome	Bortezomib carfilzomib ixazomib	Multiple myeloma, Mantle cell lymphoma	FDA-approved	[137,138,141]
Proteasome	marizomib oprozomib delanzomib	Haematological malignancies, solid tumors	Early-phase clinical trials	[142]

Alternative strategies for the development of chemotherapeutic drugs involve blocking the catalytic activity of enzymes such as E2, E3, and DUBs, or disrupting the interaction between these enzymes and specific substrates. E3 ligases and DUBs are of critical importance to the maintenance of replication fork stability (Tables 1 and 2) and are aberrantly expressed in several types of cancer (Table 4). Several small-molecule inhibitors targeting these enzymes have now been designed to inhibit cancer cell proliferation (Table 5). Inhibition of RAD18/RAD6 activity seems to represent yet another promising approach: RAD18/RAD6 activity regulates the ubiquitination of PCNA and FANCI/FANCD and is therefore required for both the DDT and FA pathways. Moreover, aberrant overexpression of RAD18 is common across a broad spectrum of cancers, including esophageal cancer [143] and gastric cancer (GC) [144] (Table 4). In GC, RAD18 depletion suppresses cell proliferation and invasiveness and increases cisplatin sensitivity [144]. In addition, SMI#9, a RAD6-selective inhibitor, attenuates chemotherapy agent-induced PCNA monoubiquitination and enhances the chemosensitivity of triple-negative breast cancer (TNBC) [145]. Notably, the development of a nanotherapy-mediated RAD6 inhibitor (SMI#9-GNP) has overcome the solubility limitations of SMI#9 [146]. Meanwhile, as the role of CRL4^Cdt2^ in origin relicensing and rereplication becomes increasingly apparent, MLN4924, a NEDD8-activating enzyme inhibitor, has been used to inhibit CRL4^Cdt2^ activity in ovarian cancer cells. The resulting persistence of the DNA replication licensing factor CDT1 at replication origins suggests that this E3 ligase may constitute a novel target in cancer therapy [147]. Other E3 inhibitors, such as MDM2 inhibitors, are currently under investigation in clinical studies (Table 5) [148].

**Table 4:** Aberrant expression of E3 ligases and DUBs regulating replication fork stability in cancers

Gene	Cancer type	Observed alterations	REF.
*MDM2*	Gastric cancer	Gene amplification	[149]
	Prostate cancer	Overexpression	[150]
	Non-small cell lung cancer	Gene polymorphisms	[151]
	Leukemias and lymphomas	Gene amplification	[152]
*RAD18*	Esophageal cancer	Overexpression	[143]
	Ovarian cancer	Overexpression	[153]
	Glioma	Overexpression	[154]
	Cervical cancer	Overexpression	[155]
	Melanoma	Overexpression	[156]
	Triple-negative breast cancer	Overexpression	[157]
*SPOP*	Prostate cancer	Gene mutation and reduced expression	[158]
	Non-small cell lung cancer	Reduced expression	[159]
	Colorectal cancer	Reduced expression	[160]
	Endometrial cancer	Gene mutation	[161]
	Kidney cancer	Overexpression	[162]
*RFWD3*	Bladder cancer	Overexpression	[163]
	Gastric cancer	Overexpression	[164]
	Colorectal cancer	Overexpression	[165]
	Non-small cell lung cancer	Overexpression	[166]
	Hepatocellular carcinoma	Overexpression	[167]
*TRAIP*	Liver cancer	Overexpression	[168]
	Osteosarcoma	Overexpression	[169]
	Lung cancer	Overexpression	[170]
	Triple-negative breast cancer	Overexpression	[171]
*CRL4^Cdt2^*	Hepatocellular carcinomas	Overexpression	[172]
	Breast cancer	Overexpression	[173]
	Gastric cancers	Overexpression	[174]
*USP1*	Breast cancer	Overexpression	[175]
	Lung cancer	Gene mutation	[176]
	Non-small cell lung cancer	Overexpression	[177]
	Cervical cancer	Overexpression	[176]
	Gastric cancer	Overexpression	[176]
	Hepatocellular carcinoma	Overexpression	[178]
	Osteosarcoma	Overexpression	[179]
	Multiple myeloma	Overexpression	[180]
	Glioblastoma	Overexpression	[181]
*USP7*	Prostate cancer	Overexpression	[182]
	Hepatocellular carcinoma	Overexpression	[161]
	Ovarian cancer	Overexpression	[183]
*USP36*	Ovarian cancer	Overexpression	[184]

**Table 5:** Potential inhibitors targeting replication fork stability for chemotherapy

Target	Inhibitor	Cancer types	Phase	REF.
RAD6	SMI#9, SMI#9-GNP	Triple-negative breast cancer	Preclinical studies	[145,146]
	TZ9	Ovarian cancer		[185]
CRL4^Cdt2^	MLN4924	Ovarian cancer	Early-phase clinical trials	[147]
MDM2	RG7112	Neuroblastoma	Early-phase clinical trials	[186]
	AMG-232, Idasanutlin	Multiple myeloma, glioblastoma		[148]
SPOP	6b	Kidney cancer	Preclinical studies	[187]
UBE2T/FANCL	CU2	Bladder cancer, lung cancer	Preclinical studies	[188]
USP1/UAF1	SJB3-019A	Multiple myeloma	Preclinical studies	[189]
	Pimozide, GW7647	Non-small cell lung cancer		[190]
	ML323	Non-small cell lung cancer, osteosarcoma		[191]
USP7	P5091, HBX41108	Colorectal cancer	Preclinical studies	[159,192]
	GNE-6776	Triple-negative breast cancer		[193]

USP1 is a well-characterized DUB that regulates the FA and TLS pathways in partnership with UAF1. It is aberrantly expressed in various types of cancer, such as non-small cell lung cancer (NSCLC), MM, osteosarcoma, and glioblastoma, where it influences cancer development and progression (Table 4) [177, 179, 181, 189]. Treatment of MM cells with USP1 inhibitor SJB3-019A leads to increased levels of Ub-FANCD2, Ub-FANCI and Ub-PCNA, and decreased RAD51 foci formation; blocking the FA pathway and inhibiting HR repair. Moreover, SJB3-019A triggers synergistic anti-MM activity when combined with other anti-MM agents, such as bortezomib, ACY1215 (an HDAC6 inhibitor), lenalidomide, or pomalidomide [189]. Additionally, the USP1/UAF1 inhibitors pimozide and GW7647 exert synergetic activity with cisplatin in cisplatin-resistant NSCLC cells (H596, NCI), but not in cisplatin-sensitive NSCLC cells (H460, NCI) [190], which may be a viable means of overcoming drug resistance. Another USP1/UAF1 inhibitor, ML323, has also been developed to enhance the cytotoxicity of cisplatin in NSCLC and osteosarcoma cells [191]. Several additional small-molecule inhibitors of E3 ligases and DUBs are also being investigated in preclinical studies [163, 166, 184].

However, intervention to the proteasome basically disrupt the turnover of the entire cellular proteome, and targeting the activities of E3 ligases or DUBs also faces specificity problems due to their multiple substrates. Development of the proteolysis-targeting chimeras (PROTACs) and deubiquitinase-targeting chimeras (DUBTACs) systems seems promising and more specific. PROTACs consists of an E3 ligand, a protein of interest, and a linker to form a ternary complex, followed by the proteasome-mediated degradation of ubiquitinated protein [194]. This technique has potential advantages in overcoming drug resistance and targeting nonenzymatic proteins compared with small molecular inhibitors. With advent of the first PROTAC bifunctional molecule for the degradation of methionine aminopeptidase 2 (MetAP-2) in 2001 [195], different PROTACs have been designed and synthesized for degradation of targeted protein, such as CDK, transcription factors, and BET protein family [196]. Moreover, oral PROTACs (ARV-110 and ARV-471) have entered clinical trial of prostate and breast cancer treatment [197,198]. Given that deubiquitination and stability of tumor suppressors or oncoproteins play important roles in carcinogenesis, DUBTACs also potentially to be a potent therapeutic intervention. For example, A DUBTAC consisting of EN523 OTUB1 recruiter and lumacaftor stabilizes ΔF508-CFTR, which improves cystic fibrosis [199]. Although still in the initial stage, the prospective PROTACs and DUBTACs molecules are attracting more attention and promising in cancer treatment. Considering the intimate relationship between replication fork stability and carcinogenesis, specific targeting of the key regulator’s stability may be promising in cancer treatment. If we can further improve our understanding of the molecular machinery that stabilizes replication forks, further novel targets for treating cancer and alleviating drug resistance are likely to emerge.

## Conclusions

DNA replication forks are extremely vulnerable to a range of endogenous and exogenous stressors. To cope with such frequent insults, which force replication forks to halt, organisms have evolved sophisticated and highly conserved mechanisms to sustain replication fork stability while the threats are resolved. Post-translational modifications, including ubiquitination, play critical roles in organizing the replication stress response machinery. Global responses triggered by ubiquitinated RPA-mediated ATR activation and ubiquitinated PCNA-mediated fork reversal/lesion bypass constitute a well-coordinated signaling network that maintains replication fork stability [12,24]. PCNA undergoes polyubiquitination following monoubiquitination by RAD18/RAD6, and PCNA mono- and polyubiquitination are essential for the coordination of fork reversal, TLS, and TS at stalled replication forks [27,84–87]. RAD18/RAD6 activity is also required for the efficient monoubiquitination of the FA core components FANCD2 and FANCI, indicating their potential involvement in resolving ICLs [133,134]. Intricate cross-talk between different signaling pathways maintains the balance of this system, and ensures that the appropriate responses are mounted to counteract replication stress under various circumstances.

Ubiquitination is a common modification that is involved in almost all cellular activities. Harnessing the ubiquitin system as a means to treat cancer is currently the focus of intensive study, and three proteasome inhibitors (bortezomib, carfilzomib, and ixazomib) have been approved by the FDA for the treatment of MM and MLC [142]. More inhibitors are currently undergoing early-phase clinical trials and preclinical studies. However, a lack of target specificity could lead to indiscriminate toxicity in both malignant and normal tissues. More specific PROTACs and DUBTACs techniques are promising in the chemotherapeutic development. Oncogenic activation itself is also known to result in the dysregulation of DNA replication, making replication stress a hallmark of cancer. The administration of chemotherapeutic drugs, such as PARP inhibitors and platinum drugs, also induces severe replication stress, providing an additional target for drug synergism. For example, kinase inhibitors targeting ATR-CHK1-WEE1 have shown promising results in combination with other chemotherapy drugs, and can overcome PARP inhibitor resistance [200].

The involvement of the ubiquitination system in the replication stress response also offers potential targets for chemotherapeutic drugs. In particular, the multiple roles of RFWD3 and RAD18 in stabilizing stalled replication forks suggests that these proteins represent candidate targets for chemotherapeutic drug development. Indeed, both RFWD3 and RAD18 are aberrantly overexpressed in a broad spectrum of cancers. Further investigation into the mechanisms underlying replication fork stabilization will facilitate a more comprehensive understanding of susceptibility and resistance when developing novel chemotherapeutic strategies.

## Abbreviations

ATM: ataxia telangiectasia mutated
ATR: ataxia-telangiectasia and RAD3-related kinase
CDC48: cell division control protein 48
CDT1: CDC10-dependent transcript 1
CHK1: checkpoint kinase 1
CMG: Cdc45-MCM-GINS
CtIP: C-terminal binding protein interacting protein
DDB1: DNA damage-binding protein 1
DPC: DNA–protein cross-link
DUBTACs: deubiquitinase-targeting chimeras
ETAA1: Ewing’s tumor-associated antigen 1
EXO1: exonuclease 1
FA: Fanconi anemia
FBH1: F-box DNA helicase 1
GC: gastric cancer
HLTF: helicase-like transcription factor
HR: homologous recombination
ICL: DNA interstrand cross-link
MCL: mantle cell lymphoma
MCM2-7: minichromosome maintenance 2-7
MM: multiple myeloma
MRE11: meiotic recombination 11 homolog 1
NSCLC: non-small cell lung cancer
ORC: origin recognition complex
PCNA: proliferating cell nuclear antigen
PRIMPOL: primase/polymerase
PROTACs: proteolysis-targeting chimeras
RPA: replication protein A
SCF: SKP1-cullin1-F-box protein
SHPRH: SNF2 histone-linker PHD-finger RING-finger helicase
SMARCAL1: SWI/SNF related matrix associated actin dependent regulator of chromatin subfamily a like 1
TIPIN: TIMELESS-interacting protein
TLS: translesion synthesis
TNBC: triple-negative breast cancer
TopBP1: DNA topoisomerase 2-binding protein 1
TS: template-switching
UPS: ubiquitin–proteasome system
USP7: ubiquitin carboxyl-terminal hydrolase 7
WRNIP1: werner helicase interacting protein 1
ZRANB3: zinc finger RANBP2-type containing 3

## References

[1] Zeman M.K. and Cimprich K.A. (2014) Causes and consequences of replication stress. Nat. Cell Biol. 16, 2–9 10.1038/ncb2897 24366029 PMC4354890

[2] Zhang B.N., Bueno Venegas A., Hickson I.D. and Chu W.K. (2019) DNA replication stress and its impact on chromosome segregation and tumorigenesis. Semin. Cancer Biol. 55, 61–69 10.1016/j.semcancer.2018.04.005 29692334

[3] Qiu S., Jiang G., Cao L. and Huang J. (2021) Replication fork reversal and protection. Front Cell Dev. Biol. 9, 670392 10.3389/fcell.2021.670392 34041245 PMC8141627

[4] Bi X. (2015) Mechanism of DNA damage tolerance. World J. Biol. Chem. 6, 48–56 10.4331/wjbc.v6.i3.48 26322163 PMC4549768

[5] Saldivar J.C., Cortez D. and Cimprich K.A. (2017) The essential kinase ATR: ensuring faithful duplication of a challenging genome. Nat. Rev. Mol. Cell Biol. 18, 622–636 10.1038/nrm.2017.67 28811666 PMC5796526

[6] Hershko A. and Ciechanover A. (1998) The ubiquitin system. Annu. Rev. Biochem. 67, 425–479 10.1146/annurev.biochem.67.1.425 9759494

[7] Yang Q., Zhao J., Chen D. and Wang Y. (2021) E3 ubiquitin ligases: styles, structures and functions. Mol. Biomed. 2, 23 10.1186/s43556-021-00043-2 35006464 PMC8607428

[8] Eletr Z.M. and Wilkinson K.D. (2014) Regulation of proteolysis by human deubiquitinating enzymes. Biochim. Biophys. Acta 1843, 114–128 10.1016/j.bbamcr.2013.06.027 23845989 PMC3833951

[9] Swatek K.N. and Komander D. (2016) Ubiquitin modifications. Cell Res. 26, 399–422 10.1038/cr.2016.39 27012465 PMC4822133

[10] Lim K.L. and Lim G.G. (2011) K63-linked ubiquitination and neurodegeneration. Neurobiol. Dis. 43, 9–16 10.1016/j.nbd.2010.08.001 20696248

[11] Tracz M. and Bialek W. (2021) Beyond K48 and K63: non-canonical protein ubiquitination. Cell. Mol. Biol. Lett. 26, 1 10.1186/s11658-020-00245-6 33402098 PMC7786512

[12] Maréchal A., Li J.M., Ji X.Y., Wu C.S., Yazinski S.A., Nguyen H.D. et al. (2014) PRP19 transforms into a sensor of RPA-ssDNA after DNA damage and drives ATR activation via a ubiquitin-mediated circuitry. Mol. Cell. 53, 235–246 10.1016/j.molcel.2013.11.002 24332808 PMC3946837

[13] Vujanovic M., Krietsch J., Raso M.C., Terraneo N., Zellweger R., Schmid J.A. et al. (2017) Replication fork slowing and reversal upon DNA damage require PCNA polyubiquitination and ZRANB3 DNA translocase activity. Mol. Cell. 67, 882.e5–90.e5 10.1016/j.molcel.2017.08.010 28886337 PMC5594246

[14] Ciccia A., Nimonkar A.V., Hu Y., Hajdu I., Achar Y.J., Izhar L. et al. (2012) Polyubiquitinated PCNA recruits the ZRANB3 translocase to maintain genomic integrity after replication stress. Mol. Cell. 47, 396–409 10.1016/j.molcel.2012.05.024 22704558 PMC3613862

[15] Leung W., Baxley R.M., Moldovan G.L. and Bielinsky A.K. (2018) Mechanisms of DNA damage tolerance: post-translational regulation of PCNA. Genes (Basel) 10, 10.3390/genes10010010 30586904 PMC6356670

[16] Coulombe P., Nassar J., Peiffer I., Stanojcic S., Sterkers Y., Delamarre A. et al. (2019) The ORC ubiquitin ligase OBI1 promotes DNA replication origin firing. Nat. Commun. 10, 2426 10.1038/s41467-019-10321-x 31160578 PMC6547688

[17] Nishitani H., Sugimoto N., Roukos V., Nakanishi Y., Saijo M., Obuse C. et al. (2006) Two E3 ubiquitin ligases, SCF-Skp2 and DDB1-Cul4, target human Cdt1 for proteolysis. EMBO J. 25, 1126–1136 10.1038/sj.emboj.7601002 16482215 PMC1409712

[18] Ma J., Shi Q., Cui G., Sheng H., Botuyan M.V., Zhou Y. et al. (2021) SPOP mutation induces replication over-firing by impairing Geminin ubiquitination and triggers replication catastrophe upon ATR inhibition. Nat. Commun. 12, 5779 10.1038/s41467-021-26049-6 34599168 PMC8486843

[19] Maculins T., Nkosi P.J., Nishikawa H. and Labib K. (2015) Tethering of SCF(Dia2) to the replisome promotes efficient ubiquitylation and disassembly of the CMG helicase. Curr. Biol. 25, 2254–2259 10.1016/j.cub.2015.07.012 26255844 PMC4562905

[20] Maric M., Mukherjee P., Tatham M.H., Hay R. and Labib K. (2017) Ufd1-Npl4 recruit Cdc48 for disassembly of ubiquitylated CMG helicase at the end of chromosome replication. Cell Rep. 18, 3033–3042 10.1016/j.celrep.2017.03.020 28355556 PMC5382235

[21] Dewar J.M., Low E., Mann M., Räschle M. and Walter J.C. (2017) CRL2(Lrr1) promotes unloading of the vertebrate replisome from chromatin during replication termination. Genes Dev. 31, 275–290 10.1101/gad.291799.116 28235849 PMC5358724

[22] Sonneville R., Moreno S.P., Knebel A., Johnson C., Hastie C.J., Gartner A. et al. (2017) CUL-2(LRR-1) and UBXN-3 drive replisome disassembly during DNA replication termination and mitosis. Nat. Cell Biol. 19, 468–479 10.1038/ncb3500 28368371 PMC5410169

[23] Roseaulin L.C., Noguchi C., Martinez E., Ziegler M.A., Toda T. and Noguchi E. (2013) Coordinated degradation of replisome components ensures genome stability upon replication stress in the absence of the replication fork protection complex. PLos Genet. 9, e1003213 10.1371/journal.pgen.1003213 23349636 PMC3547854

[24] Gong Z. and Chen J. (2011) E3 ligase RFWD3 participates in replication checkpoint control. J. Biol. Chem. 286, 22308–22313 10.1074/jbc.M111.222869 21504906 PMC3121377

[25] Gallina I., Hendriks I.A., Hoffmann S., Larsen N.B., Johansen J., Colding-Christensen C.S. et al. (2021) The ubiquitin ligase RFWD3 is required for translesion DNA synthesis. Mol. Cell. 81, 442.e9–458.e9 10.1016/j.molcel.2020.11.029 33321094 PMC7864614

[26] Herold S., Hock A., Herkert B., Berns K., Mullenders J., Beijersbergen R. et al. (2008) Miz1 and HectH9 regulate the stability of the checkpoint protein, TopBP1. EMBO J. 27, 2851–2861 10.1038/emboj.2008.200 18923429 PMC2580782

[27] Huttner D. and Ulrich H.D. (2008) Cooperation of replication protein A with the ubiquitin ligase Rad18 in DNA damage bypass. Cell Cycle 7, 3629–3633 10.4161/cc.7.23.7166 19029798

[28] Terai K., Abbas T., Jazaeri A.A. and Dutta A. (2010) CRL4(Cdt2) E3 ubiquitin ligase monoubiquitinates PCNA to promote translesion DNA synthesis. Mol. Cell. 37, 143–149 10.1016/j.molcel.2009.12.018 20129063 PMC2818832

[29] Chu W.K., Payne M.J., Beli P., Hanada K., Choudhary C. and Hickson I.D. (2015) FBH1 influences DNA replication fork stability and homologous recombination through ubiquitylation of RAD51. Nat. Commun. 6, 5931 10.1038/ncomms6931 25585578

[30] Schmid J.A., Berti M., Walser F., Raso M.C., Schmid F., Krietsch J. et al. (2018) Histone ubiquitination by the DNA damage response is required for efficient DNA replication in unperturbed S Phase. Mol. Cell. 71, 897.e8–910.e8 10.1016/j.molcel.2018.07.011 30122534

[31] Lin C.Y., Wu M.Y., Gay S., Marjavaara L., Lai M.S., Hsiao W.C. et al. (2014) H2B mono-ubiquitylation facilitates fork stalling and recovery during replication stress by coordinating Rad53 activation and chromatin assembly. PLoS Genet. 10, e1004667 10.1371/journal.pgen.1004667 25275495 PMC4183429

[32] Hung S.H., Wong R.P., Ulrich H.D. and Kao C.F. (2017) Monoubiquitylation of histone H2B contributes to the bypass of DNA damage during and after DNA replication. Proc. Natl. Acad. Sci. U.S.A. 114, E2205–E2214 10.1073/pnas.1612633114 28246327 PMC5358361

[33] Giansanti C., Manzini V., Dickmanns A., Dickmanns A., Palumbieri M.D., Sanchi A. et al. (2022) MDM2 binds and ubiquitinates PARP1 to enhance DNA replication fork progression. Cell Rep. 39, 110879 10.1016/j.celrep.2022.110879 35649362

[34] Jeong S.Y., Hariharasudhan G., Kim M.J., Lim J.Y., Jung S.M., Choi E.J. et al. (2022) SIAH2 regulates DNA end resection and replication fork recovery by promoting CtIP ubiquitination. Nucleic Acids Res. 50, 10469–10486 10.1093/nar/gkac808 36155803 PMC9561274

[35] Larsen N.B., Gao A.O., Sparks J.L., Gallina I., Wu R.A., Mann M. et al. (2019) Replication-coupled DNA-protein crosslink repair by SPRTN and the proteasome in Xenopus egg extracts. Mol. Cell. 73, 574.e7–588.e7 10.1016/j.molcel.2018.11.024 30595436 PMC6375733

[36] Wu R.A., Semlow D.R., Kamimae-Lanning A.N., Kochenova O.V., Chistol G., Hodskinson M.R. et al. (2019) TRAIP is a master regulator of DNA interstrand crosslink repair. Nature 567, 267–272 10.1038/s41586-019-1002-0 30842657 PMC6417926

[37] Deng L., Wu R.A., Sonneville R., Kochenova O.V., Labib K., Pellman D. et al. (2019) Mitotic CDK promotes replisome disassembly, fork breakage, and complex DNA rearrangements. Mol. Cell. 73, 915.e6–29.e6 10.1016/j.molcel.2018.12.021 30849395 PMC6410736

[38] Knipscheer P., Räschle M., Smogorzewska A., Enoiu M., Ho T.V., Schärer O.D. et al. (2009) The Fanconi anemia pathway promotes replication-dependent DNA interstrand cross-link repair. Science 326, 1698–1701 10.1126/science.1182372 19965384 PMC2909596

[39] Liang Z., Liang F., Teng Y., Chen X., Liu J., Longerich S. et al. (2019) Binding of FANCI-FANCD2 complex to RNA and R-loops stimulates robust FANCD2 monoubiquitination. Cell Rep. 26, 564.e5–72.e5 10.1016/j.celrep.2018.12.084 30650351 PMC6350941

[40] Yan Y., Xu Z., Huang J., Guo G., Gao M., Kim W. et al. (2020) The deubiquitinase USP36 Regulates DNA replication stress and confers therapeutic resistance through PrimPol stabilization. Nucleic Acids Res. 48, 12711–12726 10.1093/nar/gkaa1090 33237263 PMC7736794

[41] Huang T.T., Nijman S.M., Mirchandani K.D., Galardy P.J., Cohn M.A., Haas W. et al. (2006) Regulation of monoubiquitinated PCNA by DUB autocleavage. Nat. Cell Biol. 8, 339–347 10.1038/ncb1378 16531995

[42] Cohn M.A., Kowal P., Yang K., Haas W., Huang T.T., Gygi S.P. et al. (2007) A UAF1-containing multisubunit protein complex regulates the Fanconi anemia pathway. Mol. Cell. 28, 786–797 10.1016/j.molcel.2007.09.031 18082604

[43] Oestergaard V.H., Langevin F., Kuiken H.J., Pace P., Niedzwiedz W., Simpson L.J. et al. (2007) Deubiquitination of FANCD2 is required for DNA crosslink repair. Mol. Cell. 28, 798–809 10.1016/j.molcel.2007.09.020 18082605 PMC2148256

[44] Longerich S., San Filippo J., Liu D. and Sung P. (2009) FANCI binds branched DNA and is monoubiquitinated by UBE2T-FANCL. J. Biol. Chem. 284, 23182–23186 10.1074/jbc.C109.038075 19589784 PMC2749091

[45] Van Twest S., Murphy V.J., Hodson C., Tan W., Swuec P., O'rourke J.J. et al. (2017) Mechanism of ubiquitination and deubiquitination in the Fanconi anemia pathway. Mol. Cell. 65, 247–259 10.1016/j.molcel.2016.11.005 27986371

[46] Park J.M., Yang S.W., Yu K.R., Ka S.H., Lee S.W., Seol J.H. et al. (2014) Modification of PCNA by ISG15 plays a crucial role in termination of error-prone translesion DNA synthesis. Mol. Cell. 54, 626–638 10.1016/j.molcel.2014.03.031 24768535

[47] Zhao S., Kieser A., Li H.Y., Reinking H.K., Weickert P., Euteneuer S. et al. (2021) A ubiquitin switch controls autocatalytic inactivation of the DNA-protein crosslink repair protease SPRTN. Nucleic Acids Res. 49, 902–915 10.1093/nar/gkaa1224 33348378 PMC7826251

[48] Perry M., Biegert M., Kollala S.S., Mallard H., Su G., Kodavati M. et al. (2021) USP11 mediates repair of DNA-protein cross-links by deubiquitinating SPRTN metalloprotease. J. Biol. Chem. 296, 100396 10.1016/j.jbc.2021.100396 33567341 PMC7960550

[49] Huang J., Zhou Q., Gao M., Nowsheen S., Zhao F., Kim W. et al. (2020) Tandem deubiquitination and acetylation of SPRTN promotes DNA-protein crosslink repair and protects against aging. Mol. Cell. 79, 824.e5–35.e5 10.1016/j.molcel.2020.06.027 32649882 PMC7484104

[50] Bleichert F., Botchan M.R. and Berger J.M. (2015) Crystal structure of the eukaryotic origin recognition complex. Nature 519, 321–326 10.1038/nature14239 25762138 PMC4368505

[51] Ilves I., Petojevic T., Pesavento J.J. and Botchan M.R. (2010) Activation of the MCM2-7 helicase by association with Cdc45 and GINS proteins. Mol. Cell. 37, 247–258 10.1016/j.molcel.2009.12.030 20122406 PMC6396293

[52] Heller R.C., Kang S., Lam W.M., Chen S., Chan C.S. and Bell S.P. (2011) Eukaryotic origin-dependent DNA replication in vitro reveals sequential action of DDK and S-CDK kinases. Cell 146, 80–91 10.1016/j.cell.2011.06.012 21729781 PMC3204357

[53] Barbieri C.E., Baca S.C., Lawrence M.S., Demichelis F., Blattner M., Theurillat J.P. et al. (2012) Exome sequencing identifies recurrent SPOP, FOXA1 and MED12 mutations in prostate cancer. Nat. Genet. 44, 685–689 10.1038/ng.2279 22610119 PMC3673022

[54] Hernández-Pérez S., Cabrera E., Salido E., Lim M., Reid L., Lakhani S.R. et al. (2017) DUB3 and USP7 de-ubiquitinating enzymes control replication inhibitor Geminin: molecular characterization and associations with breast cancer. Oncogene 36, 4802–4809 10.1038/onc.2017.21 28288134

[55] Errico A., Cosentino C., Rivera T., Losada A., Schwob E., Hunt T. et al. (2009) Tipin/Tim1/And1 protein complex promotes Pol alpha chromatin binding and sister chromatid cohesion. EMBO J. 28, 3681–3692 10.1038/emboj.2009.304 19893489 PMC2775894

[56] Zhang D. and O'donnell M. (2016) The eukaryotic replication machine. Enzymes 39, 191–229 10.1016/bs.enz.2016.03.004 27241931

[57] Maric M., Maculins T., De Piccoli G. and Labib K. (2014) Cdc48 and a ubiquitin ligase drive disassembly of the CMG helicase at the end of DNA replication. Science 346, 1253596 10.1126/science.1253596 25342810 PMC4300516

[58] Moreno S.P., Bailey R., Campion N., Herron S. and Gambus A. (2014) Polyubiquitylation drives replisome disassembly at the termination of DNA replication. Science 346, 477–481 10.1126/science.1253585 25342805

[59] Jenkyn-Bedford M., Jones M.L., Baris Y., Labib K.P.M., Cannone G., Yeeles J.T.P. et al. (2021) A conserved mechanism for regulating replisome disassembly in eukaryotes. Nature 600, 743–747 10.1038/s41586-021-04145-3 34700328 PMC8695382

[60] Noguchi E., Noguchi C., Mcdonald W.H., Yates J.R. 3rd and Russell P. (2004) Swi1 and Swi3 are components of a replication fork protection complex in fission yeast. Mol. Cell. Biol. 24, 8342–8355 10.1128/MCB.24.19.8342-8355.2004 15367656 PMC516732

[61] Byun T.S., Pacek M., Yee M.C., Walter J.C. and Cimprich K.A. (2005) Functional uncoupling of MCM helicase and DNA polymerase activities activates the ATR-dependent checkpoint. Genes Dev. 19, 1040–1052 10.1101/gad.1301205 15833913 PMC1091739

[62] Brown E.J. and Baltimore D. (2000) ATR disruption leads to chromosomal fragmentation and early embryonic lethality. Genes Dev. 14, 397–402 10.1101/gad.14.4.397 10691732 PMC316378

[63] Fang Y., Tsao C.C., Goodman B.K., Furumai R., Tirado C.A., Abraham R.T. et al. (2004) ATR functions as a gene dosage-dependent tumor suppressor on a mismatch repair-deficient background. EMBO J. 23, 3164–3174 10.1038/sj.emboj.7600315 15282542 PMC514932

[64] Zou L. and Elledge S.J. (2003) Sensing DNA damage through ATRIP recognition of RPA-ssDNA complexes. Science 300, 1542–1548 10.1126/science.1083430 12791985

[65] Dubois J.C., Yates M., Gaudreau-Lapierre A., Clément G., Cappadocia L., Gaudreau L. et al. (2017) A phosphorylation-and-ubiquitylation circuitry driving ATR activation and homologous recombination. Nucleic Acids Res. 45, 8859–8872 10.1093/nar/gkx571 28666352 PMC5587784

[66] Lee J. and Dunphy W.G. (2010) Rad17 plays a central role in establishment of the interaction between TopBP1 and the Rad9-Hus1-Rad1 complex at stalled replication forks. Mol. Biol. Cell. 21, 926–935 10.1091/mbc.e09-11-0958 20110345 PMC2836973

[67] Haahr P., Hoffmann S., Tollenaere M.A., Ho T., Toledo L.I., Mann M. et al. (2016) Activation of the ATR kinase by the RPA-binding protein ETAA1. Nat. Cell Biol. 18, 1196–1207 10.1038/ncb3422 27723717

[68] Lin Y.C., Wang Y., Hsu R., Giri S., Wopat S., Arif M.K. et al. (2018) PCNA-mediated stabilization of E3 ligase RFWD3 at the replication fork is essential for DNA replication. Proc. Natl. Acad. Sci. U.S.A. 115, 13282–13287 10.1073/pnas.1814521115 30530694 PMC6310862

[69] Elia A.E., Wang D.C., Willis N.A., Boardman A.P., Hajdu I., Adeyemi R.O. et al. (2015) RFWD3-dependent ubiquitination of RPA regulates repair at stalled replication forks. Mol. Cell. 60, 280–293 10.1016/j.molcel.2015.09.011 26474068 PMC4609029

[70] Feeney L., Muñoz I.M., Lachaud C., Toth R., Appleton P.L., Schindler D. et al. (2017) RPA-mediated recruitment of the E3 ligase RFWD3 is vital for interstrand crosslink repair and human health. Mol. Cell. 66, 610.e4–21.e4 10.1016/j.molcel.2017.04.021 28575657 PMC5459755

[71] Motegi A., Sood R., Moinova H., Markowitz S.D., Liu P.P. and Myung K. (2006) Human SHPRH suppresses genomic instability through proliferating cell nuclear antigen polyubiquitination. J. Cell Biol. 175, 703–708 10.1083/jcb.200606145 17130289 PMC2064669

[72] Unk I., Hajdú I., Fátyol K., Hurwitz J., Yoon J.H., Prakash L. et al. (2008) Human HLTF functions as a ubiquitin ligase for proliferating cell nuclear antigen polyubiquitination. Proc. Natl. Acad. Sci. U.S.A. 105, 3768–3773 10.1073/pnas.0800563105 18316726 PMC2268824

[73] Joseph S.A., Taglialatela A., Leuzzi G., Huang J.W., Cuella-Martin R. and Ciccia A. (2020) Time for remodeling: SNF2-family DNA translocases in replication fork metabolism and human disease. DNA Repair (Amst.) 95, 102943 10.1016/j.dnarep.2020.102943 32971328 PMC8092973

[74] Ciccia A., Bredemeyer A.L., Sowa M.E., Terret M.E., Jallepalli P.V., Harper J.W. et al. (2009) The SIOD disorder protein SMARCAL1 is an RPA-interacting protein involved in replication fork restart. Genes Dev. 23, 2415–2425 10.1101/gad.1832309 19793862 PMC2764500

[75] Lemaçon D., Jackson J., Quinet A., Brickner J.R., Li S., Yazinski S. et al. (2017) MRE11 and EXO1 nucleases degrade reversed forks and elicit MUS81-dependent fork rescue in BRCA2-deficient cells. Nat. Commun. 8, 860 10.1038/s41467-017-01180-5 29038425 PMC5643552

[76] Berti M., Cortez D. and Lopes M. (2020) The plasticity of DNA replication forks in response to clinically relevant genotoxic stress. Nat. Rev. Mol. Cell Biol. 21, 633–651 10.1038/s41580-020-0257-5 32612242

[77] Thakar T., Leung W., Nicolae C.M., Clements K.E., Shen B., Bielinsky A.K. et al. (2020) Ubiquitinated-PCNA protects replication forks from DNA2-mediated degradation by regulating Okazaki fragment maturation and chromatin assembly. Nat. Commun. 11, 2147 10.1038/s41467-020-16096-w 32358495 PMC7195461

[78] Thangavel S., Berti M., Levikova M., Pinto C., Gomathinayagam S., Vujanovic M. et al. (2015) DNA2 drives processing and restart of reversed replication forks in human cells. J. Cell Biol. 208, 545–562 10.1083/jcb.201406100 25733713 PMC4347643

[79] Ho Y.C., Ku C.S., Tsai S.S., Shiu J.L., Jiang Y.Z., Miriam H.E. et al. (2022) PARP1 recruits DNA translocases to restrain DNA replication and facilitate DNA repair. PLoS Genet. 18, e1010545 10.1371/journal.pgen.1010545 36512630 PMC9794062

[80] Berti M., Ray Chaudhuri A., Thangavel S., Gomathinayagam S., Kenig S., Vujanovic M. et al. (2013) Human RECQ1 promotes restart of replication forks reversed by DNA topoisomerase I inhibition. Nat. Struct. Mol. Biol. 20, 347–354 10.1038/nsmb.2501 23396353 PMC3897332

[81] Bryant H.E., Petermann E., Schultz N., Jemth A.S., Loseva O., Issaeva N. et al. (2009) PARP is activated at stalled forks to mediate Mre11-dependent replication restart and recombination. EMBO J. 28, 2601–2615 10.1038/emboj.2009.206 19629035 PMC2738702

[82] Mailand N., Gibbs-Seymour I. and Bekker-Jensen S. (2013) Regulation of PCNA-protein interactions for genome stability. Nat. Rev. Mol. Cell Biol. 14, 269–282 10.1038/nrm3562 23594953

[83] Moldovan G.L., Pfander B. and Jentsch S. (2007) PCNA, the maestro of the replication fork. Cell 129, 665–679 10.1016/j.cell.2007.05.003 17512402

[84] Davies A.A., Huttner D., Daigaku Y., Chen S. and Ulrich H.D. (2008) Activation of ubiquitin-dependent DNA damage bypass is mediated by replication protein a. Mol. Cell. 29, 625–636 10.1016/j.molcel.2007.12.016 18342608 PMC2507760

[85] Hoege C., Pfander B., Moldovan G.L., Pyrowolakis G. and Jentsch S. (2002) RAD6-dependent DNA repair is linked to modification of PCNA by ubiquitin and SUMO. Nature 419, 135–141 10.1038/nature00991 12226657

[86] Lee K.Y. and Myung K. (2008) PCNA modifications for regulation of post-replication repair pathways. Mol. Cells 26, 5–11 18525240 PMC3518309

[87] Stelter P. and Ulrich H.D. (2003) Control of spontaneous and damage-induced mutagenesis by SUMO and ubiquitin conjugation. Nature 425, 188–191 10.1038/nature01965 12968183

[88] Tsutakawa S.E., Yan C., Xu X., Weinacht C.P., Freudenthal B.D., Yang K. et al. (2015) Structurally distinct ubiquitin- and sumo-modified PCNA: implications for their distinct roles in the DNA damage response. Structure 23, 724–733 10.1016/j.str.2015.02.008 25773143 PMC4394044

[89] Yang W. (2014) An overview of Y-Family DNA polymerases and a case study of human DNA polymerase η. Biochemistry 53, 2793–2803 10.1021/bi500019s 24716551 PMC4018060

[90] Hendel A., Ziv O., Gueranger Q., Geacintov N. and Livneh Z. (2008) Reduced efficiency and increased mutagenicity of translesion DNA synthesis across a TT cyclobutane pyrimidine dimer, but not a TT 6-4 photoproduct, in human cells lacking DNA polymerase eta. DNA Repair (Amst.) 7, 1636–1646 10.1016/j.dnarep.2008.06.008 18634905 PMC2656611

[91] Mohiuddin X.X.X., Kobayashi S., Keka I.S., Guilbaud G., Sale J., Narita T. et al. (2016) The role of HERC2 and RNF8 ubiquitin E3 ligases in the promotion of translesion DNA synthesis in the chicken DT40 cell line. DNA Repair (Amst.) 40, 67–76 10.1016/j.dnarep.2016.02.002 26994443 PMC5351851

[92] Bish R.A., Fregoso O.I., Piccini A. and Myers M.P. (2008) Conjugation of complex polyubiquitin chains to WRNIP1. J. Proteome Res. 7, 3481–3489 10.1021/pr800217q 18613717

[93] Saugar I., Parker J.L., Zhao S. and Ulrich H.D. (2012) The genome maintenance factor Mgs1 is targeted to sites of replication stress by ubiquitylated PCNA. Nucleic Acids Res. 40, 245–257 10.1093/nar/gkr738 21911365 PMC3245944

[94] Socha A., Yang D., Bulsiewicz A., Yaprianto K., Kupculak M., Liang C.C. et al. (2020) WRNIP1 is recruited to DNA interstrand crosslinks and promotes repair. Cell Rep. 32, 107850 10.1016/j.celrep.2020.107850 32640220 PMC7351111

[95] Leuzzi G., Marabitti V., Pichierri P. and Franchitto A. (2016) WRNIP1 protects stalled forks from degradation and promotes fork restart after replication stress. EMBO J. 35, 1437–1451 10.15252/embj.201593265 27242363 PMC4931187

[96] Kanu N., Zhang T., Burrell R.A., Chakraborty A., Cronshaw J., Dacosta C. et al. (2016) RAD18, WRNIP1 and ATMIN promote ATM signalling in response to replication stress. Oncogene 35, 4009–4019 10.1038/onc.2015.427 26549024 PMC4842010

[97] Branzei D. and Psakhye I. (2016) DNA damage tolerance. Curr. Opin. Cell Biol. 40, 137–144 10.1016/j.ceb.2016.03.015 27060551

[98] Pagès V., Bresson A., Acharya N., Prakash S., Fuchs R.P. and Prakash L. (2008) Requirement of Rad5 for DNA polymerase zeta-dependent translesion synthesis in Saccharomyces cerevisiae. Genetics 180, 73–82 10.1534/genetics.108.091066 18757916 PMC2535723

[99] Carlile C.M., Pickart C.M., Matunis M.J. and Cohen R.E. (2009) Synthesis of free and proliferating cell nuclear antigen-bound polyubiquitin chains by the RING E3 ubiquitin ligase Rad5. J. Biol. Chem. 284, 29326–29334 10.1074/jbc.M109.043885 19706603 PMC2785563

[100] Fumasoni M., Zwicky K., Vanoli F., Lopes M. and Branzei D. (2015) Error-free DNA damage tolerance and sister chromatid proximity during DNA replication rely on the Polα/Primase/Ctf4 Complex. Mol. Cell. 57, 812–823 10.1016/j.molcel.2014.12.038 25661486 PMC4352764

[101] Giannattasio M., Zwicky K., Follonier C., Foiani M., Lopes M. and Branzei D. (2014) Visualization of recombination-mediated damage bypass by template switching. Nat. Struct. Mol. Biol. 21, 884–892 10.1038/nsmb.2888 25195051 PMC4189914

[102] Szakal B. and Branzei D. (2013) Premature Cdk1/Cdc5/Mus81 pathway activation induces aberrant replication and deleterious crossover. EMBO J. 32, 1155–1167 10.1038/emboj.2013.67 23531881 PMC3630363

[103] Gritenaite D., Princz L.N., Szakal B., Bantele S.C., Wendeler L., Schilbach S. et al. (2014) A cell cycle-regulated Slx4-Dpb11 complex promotes the resolution of DNA repair intermediates linked to stalled replication. Genes Dev. 28, 1604–1619 10.1101/gad.240515.114 25030699 PMC4102767

[104] Menolfi D., Delamarre A., Lengronne A., Pasero P. and Branzei D. (2015) Essential Roles of the Smc5/6 Complex in Replication through Natural Pausing Sites and Endogenous DNA Damage Tolerance. Mol. Cell. 60, 835–846 10.1016/j.molcel.2015.10.023 26698660 PMC4691243

[105] Chiu R.K., Brun J., Ramaekers C., Theys J., Weng L., Lambin P. et al. (2006) Lysine 63-polyubiquitination guards against translesion synthesis-induced mutations. PLoS Genet. 2, e116 10.1371/journal.pgen.0020116 16789823 PMC1513265

[106] Urulangodi M., Sebesta M., Menolfi D., Szakal B., Sollier J., Sisakova A. et al. (2015) Local regulation of the Srs2 helicase by the SUMO-like domain protein Esc2 promotes recombination at sites of stalled replication. Genes Dev. 29, 2067–2080 10.1101/gad.265629.115 26443850 PMC4604347

[107] Gonzalez-Huici V., Szakal B., Urulangodi M., Psakhye I., Castellucci F., Menolfi D. et al. (2014) DNA bending facilitates the error-free DNA damage tolerance pathway and upholds genome integrity. EMBO J. 33, 327–340 10.1002/embj.201387425 24473148 PMC3983681

[108] Sarkies P., Reams C., Simpson L.J. and Sale J.E. (2010) Epigenetic instability due to defective replication of structured DNA. Mol. Cell. 40, 703–713 10.1016/j.molcel.2010.11.009 21145480 PMC3145961

[109] Jasencakova Z., Scharf A.N., Ask K., Corpet A., Imhof A., Almouzni G. et al. (2010) Replication stress interferes with histone recycling and predeposition marking of new histones. Mol. Cell. 37, 736–743 10.1016/j.molcel.2010.01.033 20227376

[110] Krejci L., Van Komen S., Li Y., Villemain J., Reddy M.S., Klein H. et al. (2003) DNA helicase Srs2 disrupts the Rad51 presynaptic filament. Nature 423, 305–309 10.1038/nature01577 12748644

[111] Pfander B., Moldovan G.L., Sacher M., Hoege C. and Jentsch S. (2005) SUMO-modified PCNA recruits Srs2 to prevent recombination during S phase. Nature 436, 428–433 10.1038/nature03665 15931174

[112] Bellí G., Colomina N., Castells-Roca L. and Lorite N.P. (2022) Post-translational modifications of PCNA: guiding for the best DNA damage tolerance choice. J. Fungi (Basel) 8, 621 10.3390/jof8060621 35736104 PMC9225081

[113] Lorenz A., Osman F., Folkyte V., Sofueva S. and Whitby M.C. (2009) Fbh1 limits Rad51-dependent recombination at blocked replication forks. Mol. Cell. Biol. 29, 4742–4756 10.1128/MCB.00471-09 19546232 PMC2725720

[114] Bacquin A., Pouvelle C., Siaud N., Perderiset M., Salomé-Desnoulez S., Tellier-Lebegue C. et al. (2013) The helicase FBH1 is tightly regulated by PCNA via CRL4(Cdt2)-mediated proteolysis in human cells. Nucleic Acids Res. 41, 6501–6513 10.1093/nar/gkt397 23677613 PMC3711418

[115] Lessel D., Vaz B., Halder S., Lockhart P.J., Marinovic-Terzic I., Lopez-Mosqueda J. et al. (2014) Mutations in SPRTN cause early onset hepatocellular carcinoma, genomic instability and progeroid features. Nat. Genet. 46, 1239–1244 10.1038/ng.3103 25261934 PMC4343211

[116] Stingele J., Bellelli R., Alte F., Hewitt G., Sarek G., Maslen S.L. et al. (2016) Mechanism and Regulation of DNA-Protein Crosslink Repair by the DNA-Dependent Metalloprotease SPRTN. Mol. Cell. 64, 688–703 10.1016/j.molcel.2016.09.031 27871365 PMC5128726

[117] Kröning A., Van Den Boom J., Kracht M., Kueck A.F., Meyer H. et al. (2022) Ubiquitin-directed AAA+ ATPase p97/VCP unfolds stable proteins crosslinked to DNA for proteolysis by SPRTN. J. Biol. Chem. 298, 101976 10.1016/j.jbc.2022.101976 35469923 PMC9127365

[118] Coleman K.E., Yin Y., Lui S.K.L., Keegan S., Fenyo D., Smith D.J. et al. (2022) USP1-trapping lesions as a source of DNA replication stress and genomic instability. Nat. Commun. 13, 1740 10.1038/s41467-022-29369-3 35365626 PMC8975806

[119] Semlow D.R. and Walter J.C. (2021) Mechanisms of vertebrate DNA interstrand cross-link repair. Annu. Rev. Biochem. 90, 107–135 10.1146/annurev-biochem-080320-112510 33882259

[120] Niraj J., Färkkilä A. and D'andrea A.D. (2019) The fanconi anemia pathway in cancer. Annu. Rev. Cancer Biol. 3, 457–478 10.1146/annurev-cancerbio-030617-050422 30882047 PMC6417835

[121] Neelsen K.J. and Lopes M. (2015) Replication fork reversal in eukaryotes: from dead end to dynamic response. Nat. Rev. Mol. Cell Biol. 16, 207–220 10.1038/nrm3935 25714681

[122] Garaycoechea J.I. and Patel K.J. (2014) Why does the bone marrow fail in Fanconi anemia? Blood 123, 26–34 10.1182/blood-2013-09-427740 24200684

[123] Huang M., Kim J.M., Shiotani B., Yang K., Zou L. and D'andrea A.D. (2010) The FANCM/FAAP24 complex is required for the DNA interstrand crosslink-induced checkpoint response. Mol. Cell. 39, 259–268 10.1016/j.molcel.2010.07.005 20670894 PMC2928996

[124] Yan Z., Delannoy M., Ling C., Daee D., Osman F., Muniandy P.A. et al. (2010) A histone-fold complex and FANCM form a conserved DNA-remodeling complex to maintain genome stability. Mol. Cell. 37, 865–878 10.1016/j.molcel.2010.01.039 20347428 PMC2847587

[125] Ceccaldi R., Sarangi P. and D'andrea A.D. (2016) The Fanconi anaemia pathway: new players and new functions. Nat. Rev. Mol. Cell Biol. 17, 337–349 10.1038/nrm.2016.48 27145721

[126] Alpi A.F., Pace P.E., Babu M.M. and Patel K.J. (2008) Mechanistic insight into site-restricted monoubiquitination of FANCD2 by Ube2t, FANCL, and FANCI. Mol. Cell. 32, 767–777 10.1016/j.molcel.2008.12.003 19111657

[127] Rajendra E., Oestergaard V.H., Langevin F., Wang M., Dornan G.L., Patel K.J. et al. (2014) The genetic and biochemical basis of FANCD2 monoubiquitination. Mol. Cell. 54, 858–869 10.1016/j.molcel.2014.05.001 24905007 PMC4051986

[128] Liang C.C., Zhan B., Yoshikawa Y., Haas W., Gygi S.P. and Cohn M.A. (2015) UHRF1 is a sensor for DNA interstrand crosslinks and recruits FANCD2 to initiate the Fanconi anemia pathway. Cell Rep. 10, 1947–1956 10.1016/j.celrep.2015.02.053 25801034 PMC4386029

[129] Klein Douwel D., Boonen R.A., Long D.T., Szypowska A.A., Räschle M., Walter J.C. et al. (2014) XPF-ERCC1 acts in Unhooking DNA interstrand crosslinks in cooperation with FANCD2 and FANCP/SLX4. Mol. Cell. 54, 460–471 10.1016/j.molcel.2014.03.015 24726325 PMC5067070

[130] Yamamoto K.N., Kobayashi S., Tsuda M., Kurumizaka H., Takata M., Kono K. et al. (2011) Involvement of SLX4 in interstrand cross-link repair is regulated by the Fanconi anemia pathway. Proc. Natl. Acad. Sci. U.S.A. 108, 6492–6496 10.1073/pnas.1018487108 21464321 PMC3080998

[131] Budzowska M., Graham T.G., Sobeck A., Waga S. and Walter J.C. (2015) Regulation of the Rev1-pol ζ complex during bypass of a DNA interstrand cross-link. EMBO J. 34, 1971–1985 10.15252/embj.201490878 26071591 PMC4547899

[132] Räschle M., Knipscheer P., Enoiu M., Angelov T., Sun J., Griffith J.D. et al. (2008) Mechanism of replication-coupled DNA interstrand crosslink repair. Cell 134, 969–980 10.1016/j.cell.2008.08.030 18805090 PMC2748255

[133] Zhang J., Zhao D., Wang H., Lin C.J. and Fei P. (2008) FANCD2 monoubiquitination provides a link between the HHR6 and FA-BRCA pathways. Cell Cycle 7, 407–413 10.4161/cc.7.3.5156 18277096

[134] Williams S.A., Longerich S., Sung P., Vaziri C. and Kupfer G.M. (2011) The E3 ubiquitin ligase RAD18 regulates ubiquitylation and chromatin loading of FANCD2 and FANCI. Blood 117, 5078–5087 10.1182/blood-2010-10-311761 21355096 PMC3109534

[135] Kim H., Yang K., Dejsuphong D. and D'andrea A.D. (2012) Regulation of Rev1 by the Fanconi anemia core complex. Nat. Struct. Mol. Biol. 19, 164–170 10.1038/nsmb.2222 22266823 PMC3280818

[136] Ali A.M., Pradhan A., Singh T.R., Du C., Li J., Wahengbam K. et al. (2012) FAAP20: a novel ubiquitin-binding FA nuclear core-complex protein required for functional integrity of the FA-BRCA DNA repair pathway. Blood 119, 3285–3294 10.1182/blood-2011-10-385963 22343915 PMC3321854

[137] Kane R.C., Bross P.F., Farrell A.T. and Pazdur R. (2003) Velcade: U.S. FDA approval for the treatment of multiple myeloma progressing on prior therapy. Oncologist 8, 508–513 10.1634/theoncologist.8-6-508 14657528

[138] Raedler L. (2015) Velcade (Bortezomib) receives 2 new FDA indications: for retreatment of patients with multiple myeloma and for first-line treatment of patients with mantle-cell lymphoma. Am. Health Drug Benefits 8, 135–140 26629279 PMC4665054

[139] Narayanan S., Cai C.Y., Assaraf Y.G., Guo H.Q., Cui Q., Wei L. et al. (2020) Targeting the ubiquitin-proteasome pathway to overcome anti-cancer drug resistance. Drug Resist. Updat. 48, 100663 10.1016/j.drup.2019.100663 31785545

[140] Buac D., Shen M., Schmitt S., Kona F.R., Deshmukh R., Zhang Z. et al. (2013) From bortezomib to other inhibitors of the proteasome and beyond. Curr. Pharm. Des. 19, 4025–4038 10.2174/1381612811319220012 23181572 PMC3657018

[141] Jayaweera S.P.E., Wanigasinghe Kanakanamge S.P., Rajalingam D. and Silva G.N. (2021) Carfilzomib: a promising proteasome inhibitor for the treatment of relapsed and refractory multiple myeloma. Front Oncol. 11, 740796 10.3389/fonc.2021.740796 34858819 PMC8631731

[142] Manasanch E.E. and Orlowski R.Z. (2017) Proteasome inhibitors in cancer therapy. Nat. Rev. Clin. Oncol. 14, 417–433 10.1038/nrclinonc.2016.206 28117417 PMC5828026

[143] Zou S., Yang J., Guo J., Su Y., He C., Wu J. et al. (2018) RAD18 promotes the migration and invasion of esophageal squamous cell cancer via the JNK-MMPs pathway. Cancer Lett. 417, 65–74 10.1016/j.canlet.2017.12.034 29306013

[144] Baatar S., Bai T., Yokobori T., Gombodorj N., Nakazawa N., Ubukata Y. et al. (2020) High RAD18 expression is associated with disease progression and poor prognosis in patients with gastric cancer. Ann. Surg. Oncol. 27, 4360–4368 10.1245/s10434-020-08518-2 32356270

[145] Haynes B., Gajan A., Nangia-Makker P. and Shekhar M.P. (2020) RAD6B is a major mediator of triple negative breast cancer cisplatin resistance: Regulation of translesion synthesis/Fanconi anemia crosstalk and BRCA1 independence. Biochim. Biophys. Acta Mol. Basis Dis. 1866, 165561 10.1016/j.bbadis.2019.165561 31639439 PMC6896319

[146] Saadat N., Liu F., Haynes B., Nangia-Makker P., Bao X., Li J. et al. (2018) Nano-delivery of RAD6/translesion synthesis inhibitor SMI#9 for triple-negative breast cancer therapy. Mol. Cancer Ther. 17, 2586–2597 10.1158/1535-7163.MCT-18-0364 30242094 PMC6279467

[147] Pan W.W., Zhou J.J., Yu C., Xu Y., Guo L.J., Zhang H.Y. et al. (2013) Ubiquitin E3 ligase CRL4(CDT2/DCAF2) as a potential chemotherapeutic target for ovarian surface epithelial cancer. J. Biol. Chem. 288, 29680–29691 10.1074/jbc.M113.495069 23995842 PMC3795265

[148] Konopleva M., Martinelli G., Daver N., Papayannidis C., Wei A., Higgins B. et al. (2020) MDM2 inhibition: an important step forward in cancer therapy. Leukemia 34, 2858–2874 10.1038/s41375-020-0949-z 32651541

[149] Günther T., Schneider-Stock R., Häckel C., Kasper H.U., Pross M., Hackelsberger A. et al. (2000) Mdm2 gene amplification in gastric cancer correlation with expression of Mdm2 protein and p53 alterations. Mod. Pathol. 13, 621–626 10.1038/modpathol.3880107 10874665

[150] Khor L.Y., Desilvio M., Al-Saleem T., Hammond M.E., Grignon D.J., Sause W. et al. (2005) MDM2 as a predictor of prostate carcinoma outcome: an analysis of Radiation Therapy Oncology Group Protocol 8610. Cancer 104, 962–967 10.1002/cncr.21261 16007688

[151] Luan L., Wang H., Zhao B., Wang F., Shi J. and Xu X. (2019) Association of MDM2 gene SNP 309 polymorphism and human non-small cell lung cancer susceptibility: A meta-analysis. Pathol. Res. Pract. 215, 152538 10.1016/j.prp.2019.152538 31326197

[152] Watanabe T., Ichikawa A., Saito H. and Hotta T. (1996) Overexpression of the MDM2 oncogene in leukemia and lymphoma. Leuk. Lymphoma 21, 391–397, color plates XVI following 5 10.3109/10428199609093436 9172803

[153] Zeng X., Zheng W., Sheng Y. and Ma H. (2022) UBE2B promotes ovarian cancer growth via promoting RAD18 mediated ZMYM2 monoubiquitination and stabilization. Bioengineered 13, 8000–8012 10.1080/21655979.2022.2048991 35313791 PMC9161992

[154] Sun J., Li J., Lu Z., Chen L. and Ma J. (2022) Analysis of the mechanism of RAD18 in glioma. NeuroImmunoModulation 29, 327–337 10.1159/000520761 35367987

[155] Lou P., Zou S., Shang Z., He C., Gao A., Hou S. et al. (2019) RAD18 contributes to the migration and invasion of human cervical cancer cells via the interleukin-1β pathway. Mol. Med. Rep. 20, 3415–3423 10.3892/mmr.2019.10564 31432163

[156] Wong R.P., Aguissa-Touré A.H., Wani A.A., Khosravi S., Martinka M., Martinka M. et al. (2012) Elevated expression of Rad18 regulates melanoma cell proliferation. Pigment Cell Melanoma Res. 25, 213–218 10.1111/j.1755-148X.2011.00948.x 22145991

[157] Yan X., He Y., Yang S., Zeng T., Hua Y., Bao S. et al. (2022) A positive feedback loop: RAD18-YAP-TGF-β between triple-negative breast cancer and macrophages regulates cancer stemness and progression. Cell Death Discov. 8, 196 10.1038/s41420-022-00968-9 35413945 PMC9005530

[158] García-Flores M., Casanova-Salas I., Rubio-Briones J., Calatrava A., Domínguez-Escrig J., Rubio L. et al. (2014) Clinico-pathological significance of the molecular alterations of the SPOP gene in prostate cancer. Eur. J. Cancer 50, 2994–3002 10.1016/j.ejca.2014.08.009 25204806

[159] Li J.J., Zhang J.F., Yao S.M., Huang H., Zhang S., Zhao M. et al. (2017) Decreased expression of speckle-type POZ protein for the prediction of poor prognosis in patients with non-small cell lung cancer. Oncol. Lett. 14, 2743–2748 10.3892/ol.2017.6567 28927035 PMC5588111

[160] Xu J., Wang F., Jiang H., Jiang Y., Chen J. and Qin J. (2015) Properties and clinical relevance of Speckle-Type POZ protein in human colorectal cancer. J. Gastrointest. Surg. 19, 1484–1496 10.1007/s11605-015-2767-6 26022775

[161] Cai J.B., Shi G.M., Dong Z.R., Ke A.W., Ma H.H., Gao Q. et al. (2015) Ubiquitin-specific protease 7 accelerates p14(ARF) degradation by deubiquitinating thyroid hormone receptor-interacting protein 12 and promotes hepatocellular carcinoma progression. Hepatology 61, 1603–1614 10.1002/hep.27682 25557975

[162] Zhao W., Zhou J., Deng Z., Gao Y. and Cheng Y. (2016) SPOP promotes tumor progression via activation of β-catenin/TCF4 complex in clear cell renal cell carcinoma. Int. J. Oncol. 49, 1001–1008 10.3892/ijo.2016.3609 27572476

[163] Jiang P., Xu Z., Wu S., Sun J., Tian J. and Chen X. (2022) RFWD3 acts as a tumor promotor in the development and progression of bladder cancer. Histol. Histopathol. 38, 917–928 10.14670/HH-18-558 36458571

[164] Jia J., Yang Y., Yan T., Chen T. and Lu G. (2020) Down-regulation of RFWD3 inhibits cancer cells proliferation and migration in gastric carcinoma. Gen. Physiol. Biophys. 39, 363–371 10.4149/gpb_2020009 32902405

[165] Xu F., Xiao Z., Fan L., Ruan G., Cheng Y., Tian Y. et al. (2021) RFWD3 participates in the occurrence and development of colorectal cancer via E2F1 transcriptional regulation of BIRC5. Front Cell Dev. Biol. 9, 675356 10.3389/fcell.2021.675356 34712656 PMC8547426

[166] Zhang Y., Zhao X., Zhou Y., Wang M. and Zhou G. (2020) Identification of an E3 ligase-encoding gene RFWD3 in non-small cell lung cancer. Front Med. 14, 318–326 10.1007/s11684-019-0708-6 31571161

[167] Miao Y.D., Quan W.X., Wang J.T., Gan J., Dong X. and Zhang F. (2023) Prognostic role of ring finger and WD repeat domain 3 and immune cell infiltration in hepatocellular carcinoma. World J. Hepatol. 15, 116–122 10.4254/wjh.v15.i1.116 36744161 PMC9896504

[168] Guo Z., Zeng Y., Chen Y., Liu M., Chen S., Yao M. et al. (2020) TRAIP promotes malignant behaviors and correlates with poor prognosis in liver cancer. Biomed. Pharmacother. 124, 109857 10.1016/j.biopha.2020.109857 31972358

[169] Li M., Wu W., Deng S., Shao Z. and Jin X. (2021) TRAIP modulates the IGFBP3/AKT pathway to enhance the invasion and proliferation of osteosarcoma by promoting KANK1 degradation. Cell Death Dis. 12, 767 10.1038/s41419-021-04057-0 34349117 PMC8339131

[170] Liu Y., Fan X., Zhao Z. and Shan X. (2020) LncRNA SLC7A11-AS1 contributes to lung cancer progression through facilitating TRAIP expression by inhibiting miR-4775. Onco. Targets Ther. 13, 6295–6302 10.2147/OTT.S253082 32636648 PMC7335271

[171] Zheng Y., Jia H., Wang P., Liu L., Chen Z., Xing X. et al. (2023) Silencing TRAIP suppresses cell proliferation and migration/invasion of triple negative breast cancer via RB-E2F signaling and EMT. Cancer Gene Ther. 30, 74–84 10.1038/s41417-022-00517-7 36064576 PMC9842503

[172] Pan H.W., Chou H.Y., Liu S.H., Peng S.Y., Liu C.L. and Hsu H.C. (2006) Role of L2DTL, cell cycle-regulated nuclear and centrosome protein, in aggressive hepatocellular carcinoma. Cell Cycle 5, 2676–2687 10.4161/cc.5.22.3500 17106265

[173] Ueki T., Nishidate T., Park J.H., Lin M.L., Shimo A., Hirata K. et al. (2008) Involvement of elevated expression of multiple cell-cycle regulator, DTL/RAMP (denticleless/RA-regulated nuclear matrix associated protein), in the growth of breast cancer cells. Oncogene 27, 5672–5683 10.1038/onc.2008.186 18542055

[174] Li J., Ng E.K., Ng Y.P., Wong C.Y., Yu J., Jin H. et al. (2009) Identification of retinoic acid-regulated nuclear matrix-associated protein as a novel regulator of gastric cancer. Br. J. Cancer 101, 691–698 10.1038/sj.bjc.6605202 19672268 PMC2736823

[175] Ma A., Tang M., Zhang L., Wang B., Yang Z., Liu Y. et al. (2019) USP1 inhibition destabilizes KPNA2 and suppresses breast cancer metastasis. Oncogene 38, 2405–2419 10.1038/s41388-018-0590-8 30531833

[176] García-Santisteban I., Peters G.J., Giovannetti E. and Rodríguez J.A. (2013) USP1 deubiquitinase: cellular functions, regulatory mechanisms and emerging potential as target in cancer therapy. Mol. Cancer 12, 91 10.1186/1476-4598-12-91 23937906 PMC3750636

[177] Liu Y., Luo X., Hu H., Wang R., Sun Y., Zeng R. et al. (2012) Integrative proteomics and tissue microarray profiling indicate the association between overexpressed serum proteins and non-small cell lung cancer. PLoS ONE 7, e51748 10.1371/journal.pone.0051748 23284758 PMC3526638

[178] Zhao Y., Xue C., Xie Z., Ouyang X. and Li L. (2020) Comprehensive analysis of ubiquitin-specific protease 1 reveals its importance in hepatocellular carcinoma. Cell Prolif. 53, e12908 10.1111/cpr.12908 32951278 PMC7574869

[179] Liu J., Zhu H., Zhong N., Jiang Z., Xu L., Deng Y. et al. (2016) Gene silencing of USP1 by lentivirus effectively inhibits proliferation and invasion of human osteosarcoma cells. Int. J. Oncol. 49, 2549–2557 10.3892/ijo.2016.3752 27840911

[180] Salminen A., Suuronen T., Huuskonen J. and Kaarniranta K. (2008) NEMO shuttle: a link between DNA damage and NF-kappaB activation in progeroid syndromes? Biochem. Biophys. Res. Commun. 367, 715–718 10.1016/j.bbrc.2007.11.189 18201555

[181] Lee J.K., Chang N., Yoon Y., Yang H., Cho H., Kim E. et al. (2016) USP1 targeting impedes GBM growth by inhibiting stem cell maintenance and radioresistance. Neuro. Oncol. 18, 37–47 10.1093/neuonc/nov091 26032834 PMC4677407

[182] Song M.S., Salmena L., Carracedo A., Egia A., Lo-Coco F., Teruya-Feldstein J. et al. (2008) The deubiquitinylation and localization of PTEN are regulated by a HAUSP-PML network. Nature 455, 813–817 10.1038/nature07290 18716620 PMC3398484

[183] Ma M. and Yu N. (2016) Ubiquitin-specific protease 7 expression is a prognostic factor in epithelial ovarian cancer and correlates with lymph node metastasis. Onco. Targets Ther. 9, 1559–1569 27051296 10.2147/OTT.S100050 PMC4803273

[184] Li J., Olson L.M., Zhang Z., Li L., Bidder M., Nguyen L. et al. (2008) Differential display identifies overexpression of the USP36 gene, encoding a deubiquitinating enzyme, in ovarian cancer. Int. J. Med. Sci. 5, 133–142 10.7150/ijms.5.133 18566677 PMC2424181

[185] Somasagara R.R., Spencer S.M., Tripathi K., Clark D.W. et al. (2017) RAD6 promotes DNA repair and stem cell signaling in ovarian cancer and is a promising therapeutic target to prevent and treat acquired chemoresistance. Oncogene 36, 6680–6690 10.1038/onc.2017.279 28806395 PMC5709226

[186] Al-Ghabkari A. and Narendran A. (2019) In vitro characterization of a potent p53-MDM2 inhibitor, RG7112 in neuroblastoma cancer cell lines. Cancer Biother. Radiopharm. 34, 252–257 30724592 10.1089/cbr.2018.2732

[187] Stone L. (2016) Kidney cancer: On target - inhibiting SPOP in ccRCC. Nat. Rev. Urol. 13, 630 10.1038/nrurol.2016.195 27698399

[188] Cornwell M.J., Thomson G.J., Coates J., Belotserkovskaya R., Waddell I.D., Jackson S.P. et al. (2019) Small-molecule inhibition of UBE2T/FANCL-mediated ubiquitylation in the Fanconi anemia pathway. ACS Chem. Biol. 14, 2148–2154 10.1021/acschembio.9b00570 31525021 PMC6804243

[189] Das D.S., Das A., Ray A., Song Y., Samur M.K., Munshi N.C. et al. (2017) Blockade of deubiquitylating enzyme USP1 inhibits DNA repair and triggers apoptosis in multiple myeloma cells. Clin. Cancer Res. 23, 4280–4289 10.1158/1078-0432.CCR-16-2692 28270494 PMC5540781

[190] Chen J., Dexheimer T.S., Ai Y., Liang Q., Villamil M.A., Inglese J. et al. (2011) Selective and cell-active inhibitors of the USP1/ UAF1 deubiquitinase complex reverse cisplatin resistance in non-small cell lung cancer cells. Chem. Biol. 18, 1390–1400 10.1016/j.chembiol.2011.08.014 22118673 PMC3344384

[191] Liang Q., Dexheimer T.S., Zhang P., Rosenthal A.S., Villamil M.A., You C. et al. (2014) A selective USP1-UAF1 inhibitor links deubiquitination to DNA damage responses. Nat. Chem. Biol. 10, 298–304 10.1038/nchembio.1455 24531842 PMC4144829

[192] Colland F., Formstecher E., Jacq X., Reverdy C., Planquette C., Conrath S. et al. (2009) Small-molecule inhibitor of USP7/HAUSP ubiquitin protease stabilizes and activates p53 in cells. Mol. Cancer Ther. 8, 2286–2295 10.1158/1535-7163.MCT-09-0097 19671755

[193] Korenev G., Yakukhnov S., Druk A., Golovina A., Chasov V., Mirgayazova R. et al. (2022) USP7 inhibitors in cancer immunotherapy: current status and perspective. Cancers (Basel) 14, 10.3390/cancers14225539 36428632 PMC9688046

[194] Li X. and Song Y. (2020) Proteolysis-targeting chimera (PROTAC) for targeted protein degradation and cancer therapy. J. Hematol. Oncol. 13, 50 10.1186/s13045-020-00885-3 32404196 PMC7218526

[195] Sakamoto K.M., Kim K.B., Kumagai A., Mercurio F., Crews C.M. and Deshaies R.J. (2001) Protacs: chimeric molecules that target proteins to the Skp1-Cullin-F box complex for ubiquitination and degradation. Proc. Natl. Acad. Sci. U.S.A. 98, 8554–8559 10.1073/pnas.141230798 11438690 PMC37474

[196] Xi J.Y., Zhang R.Y., Chen K., Yao L., Li M.Q., Jiang R. et al. (2022) Advances and perspectives of proteolysis targeting chimeras (PROTACs) in drug discovery. Bioorg. Chem. 125, 105848 10.1016/j.bioorg.2022.105848 35533582

[197] Gao X., Iii H.a.B., Vuky J., Dreicer R., Sartor A.O., Sternberg C.N. et al. (2022) Phase 1/2 study of ARV-110, an androgen receptor (AR) PROTAC degrader, in metastatic castration-resistant prostate cancer (mCRPC). J. Clin. Oncol. 40, 17 10.1200/JCO.2022.40.6_suppl.017

[198] Snyder L.B., Flanagan J.J., Qian Y., Gough S.M., Andreoli M., Bookbinder M. et al. (2021) Abstract 44: The discovery of ARV-471, an orally bioavailable estrogen receptor degrading PROTAC for the treatment of patients with breast cancer. Cancer Res. 81, 44 10.1158/1538-7445.AM2021-44

[199] Henning N.J., Boike L., Spradlin J.N., Ward C.C., Liu G., Zhang E. et al. (2022) Deubiquitinase-targeting chimeras for targeted protein stabilization. Nat. Chem. Biol. 18, 412–421 10.1038/s41589-022-00971-2 35210618 PMC10125259

[200] Da Costa A., Chowdhury D., Shapiro G.I., D'andrea A.D. and Konstantinopoulos P.A. (2023) Targeting replication stress in cancer therapy. Nat. Rev. Drug Discov. 22, 38–58 10.1038/s41573-022-00558-5 36202931 PMC11132912

